# Associations between vitamin K and systemic immune and inflammation biomarkers: a population-based study from the NHANES (2007–2020)

**DOI:** 10.3389/fnut.2025.1625209

**Published:** 2025-07-11

**Authors:** Wenjiao Luo, Dong Ye, Kun Zhao, Liang Zhou, Yanfei Wu, Qiuhua Ge

**Affiliations:** Department of Rehabilitation Medicine, Zhejiang Provincial People’s Hospital (Affiliated People’s Hospital), Center for Rehabilitation Medicine, Rehabilitation and Sports Medicine Research Institute of Zhejiang Province, Hangzhou Medical College, Hangzhou, Zhejiang, China

**Keywords:** vitamin K, systemic immune inflammatory index, systemic inflammatory response index, systemic immune-inflammation response index, neutrophil-tolymphocyte ratio

## Abstract

**Background:**

With the aging of the population, finding effective interventions and treatments to delay chronic inflammation-related diseases is an urgent problem to be solved. Previous studies on animals have proposed that vitamin K can inhibit inflammation and may be a potential regulator of the immune inflammatory response. These findings suggested that increasing intake of vitamin K might also help reduce inflammation.

**Methods:**

This study included 36,895 people from the National Health and Nutrition Examination Survey (2007–2020). Multivariate linear regression and restricted cubic spline analyses were conducted to explore the association between vitamin K intake and various immune inflammatory factors. Subgroup analyses were performed based on age, gender, ethnicity, BMI, hyperlipidemia, diabetes and hypertension.

**Results:**

After multivariable adjustment, vitamin K intake is negatively correlated with SII, SIRI, SIIRI, NLR, white blood cell, neutrophil, and monocyte. When the level of vitamin K intake was less than 237.7 mcg/d, RAR showed a significant decreasing trend with the increase of vitamin K. When the vitamin K intake level was lower than 75.1 mcg/d, the basophil showed a downward trend with the increase of intake. However, when vitamin K levels exceed the inflection point, the above association no longer exists.

**Conclusion:**

These findings reveal that vitamin K intake is associated with reduced inflammatory status and improvements in immune inflammatory biomarkers. Vitamin K may modulate systemic immune and inflammatory markers, which may play a role in the development of chronic inflammation.

## 1 Introduction

Chronic inflammation is a pervasive feature of most age-related diseases, such as cardiovascular disease, osteoporosis, dementia, chronic kidney or liver disease and neurodegenerative disease (1). With advancing age, inflammation not only leads to a decline in immune response and adaptation (2), but also plays an important role in cognitive decline (3, 4). Therefore, there is an urgent need for new and more effective options to alter the dynamics of inflammation and slow inflammation-associated chronic diseases.

As a multifunctional biomolecule, vitamin K is not only involved in the coagulation process, it acts as a key coenzyme in γ-carboxylation process (5), but also participates in the glutamyl carboxylation of many proteins and can improve mitochondrial function. It may offer survival benefits in cardiovascular disease, chronic kidney disease, cancer, and bone metabolism (6–9). Vitamin K is a naphthoquinone compound and contains a variety of forms. Vitamin K3 is the simplest, but it cannot be obtained through a natural diet. Vitamin K1 is abundant in vegetables such as kale, parsley, spinach, leek, and purslane, and vitamin K2 is mostly obtained through diets such as yogurt, cheese, sauerkraut, and natto (10). Phylloquinone (vitamin K1) in serum is the most used marker of vitamin K status (11), but it is not routinely detected in clinics. Dietary intake can be used as an indirect tool to monitor the vitamin K supply.

There is limited evidence that vitamin K exerted a potent protective effect against acute lung injury in mice and liver injury in naturally aging rats by inhibiting inflammation (12, 13). In addition, Eggebrecht et al. reported that long-term vitamin K antagonist’s users had higher levels of high-sensitivity C-reactive protein compared to short-term users (14). Based on the above evidence, it is speculated that vitamin K may have a modulating effect on systemic immunity and inflammation.

Leukocytes, lymphocytes, neutrophils, monocytes, and other blood cellular markers are commonly used to assess inflammation in human nutritional studies (15). Over the last decades various cellular immune inflammation markers developed from blood cell components have gradually emerged as clinical indicators of disease-related inflammation. Systemic immune inflammatory index (SII) (16) and systemic inflammatory response index (SIRI) (17) were initially found to be positively correlated with poor prognosis for hepatocellular carcinoma and pancreatic cancer, respectively. SII and SIRI can integrate multiple leukocyte subsets and assess systemic inflammatory activity in individuals (18). An observational study indicated that an increased systemic immune-inflammation index (SIIRI) was associated with a poor cardiovascular outcome in patients initially diagnosed with coronary artery disease (CAD). In a Danish longitudinal study, it was observed that increasing neutrophil-to-lymphocyte ratio (NLR) were associated with chronic inflammation (19). The NLR and platelet-to-lymphocyte ratio (PLR) could serve as biomarkers of cancer incidence risk (20) and were also associated with post-thrombolysis early neurological outcomes (21) as well as stroke-associated pneumonia (22). As another innovative indicator of immune status and response, red blood cell distribution width-to-albumin ratio (RAR) has also been associated with age-related diseases such as heart failure (23). A population-based prospective cohort study revealed that a higher baseline RAR was linked to an elevated risk of overall and cause-specific mortality in the general population (24). In addition, ferritin and high-sensitivity C-reactive protein (hs-CRP) are classic inflammatory biomarkers widely used in clinical context. All the markers mentioned above are obtained from routine laboratory tests or can be derived through calculation.

Therefore, this study aimed to explore the association between vitamin K intake and systemic immunity and inflammation through a large representative population and various cellular immune inflammation markers.

## 2 Materials and methods

### 2.1 Study population

National Health and Nutrition Examination Survey (NHANES) is an ongoing project that conducts a demographic survey of non-institutional citizens in the United States, with data released every 2 years. The nationally representative survey includes interviews, physical examinations, and laboratory tests, which provide a wealth of information on nutrition and health. All analyses in this study were performed in accordance with NHANES guidelines and regulations. And the datasets analyzed during the current study are available at NHANES website^1^. The Ethics Review Board of the National Center for Health Statistics Research provided ethics approvals (Protocol #2005–06, #2011–17, #2018–01) for all potential study protocols in the NHANES. Written informed consent was obtained from each participant.^2^ Therefore, no additional ethical approval and informed consent is required.

**FIGURE 1:**
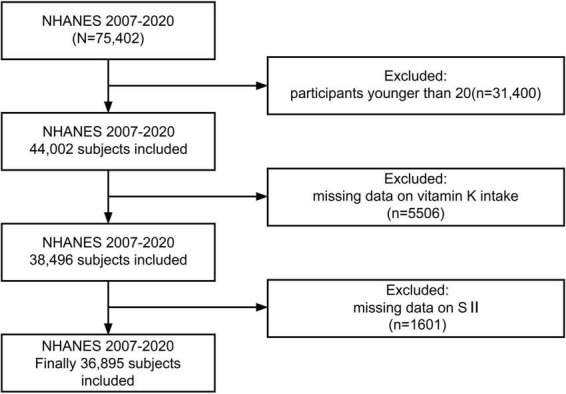
Flowchart of participant selection.

In this investigation, 7 cycles of NHANES from 2007 to 2020 were downloaded, remaining a total of 44,002 participants after excluding those younger than 20. Those with missing data on vitamin K intake (*n* = 5,506) and systemic immune-inflammation index (*n* = 1,601) were further removed, leaving 36,895 individuals for further analysis (Figure 1).

### 2.2 Assessment of vitamin K intake

The participants’ intake of vitamin K, from diet and dietary supplements, was obtained from two 24-h dietary recall interviews during the survey period (2007–2020). The first dietary recall was collected in the Mobile Examination Center (MEC) and the second interview was conducted by telephone after 3–10 days. Since 2007, all vitamins, minerals, herbals and other dietary supplements as well as non-prescription antacids have been collected in dietary interviews. A set of measuring guides (various glasses, bowls, mugs, drink boxes and bottles, household spoons, measuring cups and spoons, a ruler, thickness sticks, bean bags, and circles) was provided in the MEC dietary interview room for the participant to use when reporting food quantities. Dietary data were collected by trained interviewers using the standardized Automated Multiple-Pass Method (AMPM). The above-mentioned elements ensured that participants received sufficient support and guidance during the recall process, thus minimizing errors and omissions. Compared to the first day, the dietary recall conducted by telephone may have had limitations, leading to less detailed and accurate information. A single 24-h recall has historically been considered sufficient to describe the mean (25). Considering the standardization and high quality of the in-person interview, only the first day’s data was used in the analysis to ensure accuracy (26, 27). The vitamin K content in all foods was calculated by the United States Department of Agriculture’s Food and Nutrient Database for Dietary Studies 3.0 (FNDDS 3.0) (28, 29). Total vitamin K intake is calculated by summing intake from diet and supplements.

### 2.3 Definition of SII and other inflammatory markers

Blood specimens of all study participants were measured at the NHANES MECs. A complete blood count is performed on the CoulterDxH 800 analyzer, and the neutrophil count, lymphocyte count, monocytes count, eosinophil count, basophil count and platelet count were reported as 1,000 cell/μL. Detailed and comprehensive test methods can be found on the NHANES website (30). The calculation formulas of SII, SIRI, NLR, PLR and RAR have been described in previous studies as follows: SII = N × PLT/L (16), SIRI = N × M/L (17), SIIRI = N × M × PLT/L (31), NLR = N/L, PLR = PLT/L, RAR = [RDW(%)]/[albumin(g/dL)] (32) (where L:lymphocyte count, M: monocytes count, N: neutrophil count, PLT: platelet count(10^3^/μL), RDW: red cell distribution width). Standardized protocols for measuring these commonly used biochemical markers, including ferritin, albumin, and hs-CRP, can also be searched for on the NHANES website.

### 2.4 Selection of covariates

The covariates in the analysis included demographic characteristics [age, sex, race, education, family poverty income ratio (≤ 1.3, 1.3–3.5, ≥ 3.5), and body mass index (BMI)], lifestyle (smoking status, alcohol consumption, and vigorous recreational activity), chronic diseases (hyperlipidemia, hypertension, and diabetes).

The level of alcohol consumption is classified according to the following criteria: heavy (≥ 3 drinks per day for female and ≥ 4 drinks per day for male; or binge drinking ≥ 5 days per month), moderate (≥ 2 drink per day for female and ≥ 3 drink per day for male; or binge drinking ≥ 2 days per month), mild (≥ 1 drink per day for female and ≥ 2 drink per day for male), former (≥ 12 drinks in 1 year and did not drink last year, or did not drink last year but drank ≥ 12 drinks in lifetime), never (< 12 drinks in lifetime). Adults who smoked less than 100 cigarettes in life were considered as non-smokers and those who smoked more than 100 cigarettes were divided into former and current smokers by whether they were still smoking now. Hypertension was defined as systolic blood pressure ≥ 130 mmHg and/or diastolic blood pressure ≥ 80 mmHg by 3 measures. Participants who achieved one of the following conditions were diagnosed with hyperlipidemia: triglycerides ≥ 150 mg/dL, total cholesterol ≥ 200 mg/dL, low-density lipoprotein ≥ 130 mg/dL, high-density lipoprotein < 40 mg/dL in male (< 50 mg/dL in female), or taking lipid-lowering drugs. The diagnostic criteria for diabetes are as follows: glycohemoglobin HbA1c (%) ≥ 6.5, fasting glucose ≥ 7.0 mmol/L, random blood glucose or 2-h OGTT blood glucose ≥ 11.1 mmol/L, doctor told you have diabetes, use of diabetes medication or insulin. Participants with BMI ≤ 18.5, 18.5–25, 25–30, ≥ 30 were classified as underweight, normal, overweight and obese. Dietary inflammatory index (DII) provided a tool to compare the inflammatory potential of diets, either anti-inflammatory or pro-inflammatory (33). Who experienced vigorous recreational activities was defined according to the question “In a typical week do you do any vigorous-intensity sports, fitness, or recreational activities that cause large increases in breathing or heart rate like running or basketball for at least 10 min continuously?”.

### 2.5 Statistical analyses

All statistical analyses were conducted using the R packages survey and srvyr, accounting for the complex, multistage sampling design of NHANES, including sampling weights, stratification, and clustering. Continuous variables were expressed as survey-weighted mean (standard error) and the Wilcoxon rank-sum test was used for the comparison between groups. Categorical variables were described by unweighted frequencies (survey-weighted percentages) and Rao-Scott chi-square test was conducted for the comparison.

First, we conducted univariate analysis and multivariate linear regression to explore the association between vitamin K intake and SII, as well as other immune inflammatory factors. Vitamin K intake was converted into categorical variables by quartile, and four multiple regression models were applied in this study. The crude model was the non-adjusted model. Model 1 was slightly adjusted for age and gender. Model 2 was adjusted for age, gender, race, family poverty income ratio and educational level. Model 3 was a fully adjusted model with all confounders, including age, gender, race, family poverty income ratio, educational level, BMI, smoking status, alcohol consumption, vigorous recreational activity, dietary inflammatory index, hyperlipidemia, hypertension, and diabetes.

Next, the possibility of a non-linear relationship between vitamin K intake (continuous variable) and SII, as well as other outcomes, was explored using restricted cubic spline. If the relationship was found to be non-linear, the inflection point between vitamin K intake and inflammatory markers was calculated. Piecewise regression was then performed on both sides of the inflection point value. Then, a stratified logistic regression mode was used for subgroup analysis and interaction analysis based on age, gender, ethnicity, BMI, hyperlipidemia, diabetes and hypertension, where *p*-value was calculated by logistic regression analysis and p for interaction.

The downloaded data was visualized and analyzed using the R software version 4.3.0, and *p*-value < 0.05 was considered statistically significant.

## 3 Results

### 3.1 Baseline characteristics

Upon excluding participants younger than 20 and those without data about vitamin K intake and SII, a total of 36,895 adults were enrolled in the study and the mean age was 47.76 (0.23) years. The gender distribution was roughly equal, with 48.05% male and 51.95% female. Most participants were non-Hispanic white (*n* = 15,116, 65.98%). The descriptive characteristics of the individuals by vitamin K intake quartiles (Q1: 0–39.9 mcg/d, *n* = 9,234; Q2: 39.9–72.1 mcg/d, *n* = 9,216; Q3: 72.1–131.3 mcg/d, n = 9226; Q4:131.3–45067.1 mcg/d, *n* = 9219) are shown in Table 1, and indicators such as SII and SIRI are recorded in Table 2. Participants with higher vitamin K intake had lower levels of SII, SIRI, SIIRI, NLR, RAR, hs-CRP, white blood cell, neutrophil, lymphocyte, monocyte, eosinophil, and basophil. Groups with higher vitamin K intake also tended to have higher incomes, higher educations, and they were more likely to be never-smokers, drink less, be more physically active and enjoy more anti-inflammatory diet. There were no significant differences in the prevalence of diabetes between groups, however, higher proportions of hyperlipidemia were observed in lower vitamin K intake groups.

**TABLE 1:** Baseline characteristics of study population according to vitamin K intake level.

Characteristic	N^1^	Overall, *N* = 36,895 (100%)^2^	Q1, *N* = 9,234 (22%)^2^	Q2, *N* = 9,216 (24%)^2^	Q3, *N* = 9,226 (26%)^2^	Q4, *N* = 9,219 (27%)^2^	*P*-Value^3^
**Age**	36,895	47.76(0.23)	45.61(0.29)	47.13(0.29)	48.86(0.32)	49.03(0.36)	**<0.001**
**Gender**	36,895						**<0.001**
Female		18,962(51.95%)	5,208(57.83%)	4,671(51.29%)	4,426(48.86%)	4,657(50.62%)	
Male		17,933(48.05%)	4,026(42.17%)	4,545(48.71%)	4,800(51.14%)	4,562(49.38%)	
**Race**	36,895						**<0.001**
Non-hispanic white		15,116(65.98%)	3,532(61.82%)	3,728(64.79%)	4,017(68.01%)	3,839(68.53%)	
Non-Hispanic Black		8,057(10.96%)	2,306(14.22%)	1,948(11.23%)	1,810(9.35%)	1,993(9.56%)	
Mexican American		5,364(8.70%)	1,532(10.13%)	1,455(9.69%)	1,380(8.89%)	997(6.46%)	
Other Hispanic		3,863(6.17%)	1,054(7.11%)	1,077(6.78%)	913(5.72%)	819(5.27%)	
Other Race		4,495(8.20%)	810(6.72%)	1,008(7.50%)	1,106(8.03%)	1,571(10.18%)	
**Family PIR**	36,895						**<0.001**
≤1.3		10,461(20.40%)	3,425(29.06%)	2,686(21.37%)	2,334(18.13%)	2,016(14.61%)	
1.3–3.5		12,650(32.18%)	3,139(33.29%)	3,365(34.51%)	3,212(33.19%)	2,934(28.27%)	
> 3.5		10,259(39.83%)	1,736(29.69%)	2,276(36.43%)	2,790(40.98%)	3,457(50.04%)	
**Education**	36,895						**<0.001**
Less than 12th grade		8,408(14.69%)	2,874(21.42%)	2,251(16.11%)	1,865(13.27%)	1,418(9.26%)	
High school graduate		8,531(23.93%)	2,382(28.39%)	2,282(26.13%)	2,100(24.30%)	1,767(18.00%)	
Some college or associate degree		11,234(31.68%)	2,658(30.90%)	2,788(32.51%)	2,919(32.16%)	2,869(31.12%)	
College graduate or above		8,684(29.65%)	1,308(19.21%)	1,891(25.23%)	2,331(30.22%)	3,154(41.56%)	
**BMI**	36,531	29.21(0.08)	29.38(0.11)	29.63(0.12)	29.14(0.12)	28.77(0.13)	**<0.001**
Hyperlipidemia	36,895						**0.007**
Yes		26,072(69.96%)	6,693(72.00%)	6,476(69.07%)	6,491(70.68%)	6,412(68.40%)	
No		10,821(30.04%)	2,541(28.00%)	2,740(30.93%)	2,734(29.32%)	2,806(31.60%)	
**Hypertension**	36,895						**0.030**
Yes		18,809(47.35%)	4,764(46.46%)	4,693(47.23%)	4,784(49.39%)	4,568(46.28%)	
No		18,075(52.62%)	4,467(53.51%)	4,522(52.76%)	4,439(50.59%)	4,647(53.70%)	
**Diabetes**	36,895						0.2
Yes		7,262(14.70%)	1,893(14.97%)	1,842(15.06%)	1,826(15.38%)	1,701(13.51%)	
No		29,250(84.28%)	7,247(84.05%)	7,263(83.80%)	7,309(83.65%)	7,431(85.46%)	
**Smoke**	36,895						**<0.001**
Never		20,643(55.81%)	4,899(52.50%)	5,053(53.90%)	5,211(56.28%)	5,480(59.77%)	
Former		8,934(24.88%)	1,907(19.78%)	2,257(25.11%)	2,383(26.94%)	2,387(26.90%)	
Now		7,299(19.27%)	2,421(27.68%)	1,904(20.99%)	1,626(16.74%)	1,348(13.27%)	
**Alcohol user**	36,895						**<0.001**
Never		4,462(9.48%)	1,362(12.24%)	1,132(10.21%)	990(8.45%)	978(7.55%)	
Former		4,286(10.07%)	1,277(11.90%)	1,159(11.21%)	999(9.59%)	851(8.03%)	
Mild		11,749(34.42%)	2,344(26.67%)	2,795(31.60%)	3,125(36.74%)	3,485(41.07%)	
Moderate		5,401(16.74%)	1,299(16.59%)	1,313(15.76%)	1,372(17.17%)	1,417(17.31%)	
Heavy		6,799(20.27%)	1,879(22.83%)	1,804(22.05%)	1,634(18.69%)	1,482(18.09%)	
**VRA**	36,893	8,190(26.65%)	1,544(20.74%)	1,824(23.17%)	2,093(26.23%)	2,729(34.95%)	**<0.001**
**DII**	36,895						**<0.001**
Anti-inflammation		8,021(24.40%)	303(3.88%)	1,041(12.85%)	2,258(25.74%)	4,419(50.09%)	
Pro-inflammation		28,874(75.60%)	8,931(96.12%)	8,175(87.15%)	6,968(74.26%)	4,800(49.91%)	

^1^N not Missing (unweighted). ^2^Mean(SE); n (unweighted) (%). ^3^Wilcoxon rank-sum test for complex survey samples; chi-squared test with Rao & Scott’s second-order correction. Data are presented as unweighted counts (n) and survey-weighted percentages (%) for categorical variables, and as survey-weighted means with standard errors (SE) for continuous variables. All comparisons accounted for the NHANES complex sampling design. family PIR, family poverty income ratio; BMI, Body Mass Index; VRA, vigorous recreational activities; DII, Dietary inflammatory index. The median (range) of dietary vitamin K intakes for each quartile is as follows: Q1: 25 (0–39.9) mcg/d; Q2: 54.6(39.9–72.1) mcg/d; Q3: 95.5(72.1–131.3) mcg/d; Q4: 212.5(131.3–45067.1) mcg/d. Values in bold indicate statistically significant differences (*P* < 0.05).

**TABLE 2:** Immune inflammatory factors of study population according to vitamin K intake level.

Characteristic	N^1^	Overall, *N* = 36,895 (100%)^2^	Q1, *N* = 9,234 (22%)^2^	Q2, *N* = 9,216 (24%)^2^	Q3, *N* = 9,226 (26%)^2^	Q4, *N* = 9,219 (27%)^2^	*P*-Value^3^
**SII**	36,895	536.09 (3.36)	557.89 (5.00)	537.12 (5.63)	536.07 (4.86)	517.42 (4.92)	**<0.001**
**SIRI**	36,895	1.28 (0.01)	1.31 (0.02)	1.27 (0.02)	1.30 (0.01)	1.23 (0.01)	**<0.001**
**SIIRI**	36,895	316.39 (2.78)	335.45 (4.44)	317.03 (5.07)	318.20 (3.83)	298.57 (3.91)	**<0.001**
**NLR**	36,895	2.20 (0.01)	2.21 (0.02)	2.19 (0.02)	2.21 (0.02)	2.16 (0.02)	**0.045**
**PLR**	36,895	123.77 (0.52)	123.78 (0.79)	123.32 (0.86)	123.69 (0.79)	124.23 (0.86)	0.6
**RAR**	36,199	3.17 (0.01)	3.22 (0.01)	3.17 (0.01)	3.16 (0.01)	3.13 (0.01)	**<0.001**
**ferritin**	15,806	122.86 (1.89)	107.89 (2.79)	121.88 (2.67)	130.75 (3.31)	129.13 (3.44)	**<0.001**
**hs-CRP**	16,343	3.90 (0.10)	4.67 (0.29)	4.07 (0.15)	3.65 (0.14)	3.46 (0.21)	**<0.001**
**WBC**	36,895	7.33 (0.03)	7.61 (0.05)	7.39 (0.05)	7.31 (0.04)	7.09 (0.04)	**<0.001**
**Neut**	36,895	4.34 (0.02)	4.54 (0.04)	4.36 (0.03)	4.34 (0.03)	4.18 (0.03)	**<0.001**
**Lym**	36,895	2.17 (0.01)	2.24 (0.01)	2.22 (0.04)	2.14 (0.01)	2.11 (0.02)	**<0.001**
**MO**	36,895	0.57 (0.00)	0.58 (0.00)	0.57 (0.00)	0.57 (0.00)	0.56 (0.00)	**<0.001**
**EOS**	36,895	0.20 (0.00)	0.20 (0.00)	0.20 (0.00)	0.20 (0.00)	0.19 (0.00)	**<0.001**
**BAS**	36,895	0.05 (0.00)	0.05 (0.00)	0.05 (0.00)	0.05 (0.00)	0.05 (0.00)	**<0.001**

^1^N not Missing (unweighted), ^ 2^Mean(SE), ^3^Wilcoxon rank-sum test for complex survey samples. Data are presented as unweighted counts (n) and survey-weighted percentages (%) for categorical variables, and as survey-weighted means with standard errors (SE) for continuous variables. All comparisons accounted for the NHANES complex sampling design. SII, systemic immune-inflammation index; SIRI, systemic inflammation response index; SIIRI, systemic immune-inflammation response index; NLR, neutrophil-to-lymphocyte ratio; PLR, platelet-to-lymphocyte ratio; RAR, red blood cell distribution width-to-albumin ratio; hs-CRP, high-sensitivity C-reactive protein; WBC, white blood cell; Neut, neutrophil; Lym, lymphocyte; MO, monocyte; EOS, eosinophil; BAS, basophil. The median (range) of dietary vitamin K intakes for each quartile is as follows: Q1: 25 (0–39.9) mcg/d; Q2: 54.6(39.9–72.1) mcg/d; Q3: 95.5(72.1–131.3) mcg/d; Q4: 212.5(131.3–45067.1) mcg/d. Values in bold indicate statistically significant differences (*P* < 0.05).

### 3.2 Associations between vitamin K intake and SII, SIRI, NLR, PLR, RAR, hs-CRP, ferritin, and white blood cell

Four regression models were designed to investigate the correlation between vitamin K intake and immune inflammatory factors. Vitamin K intake is significantly negatively correlated with SII, SIRI, SIIRI, NLR, RAR, white blood cell, neutrophil, monocyte, and eosinophil in all four models (Table 3; Supplementary Table 1).

**TABLE 3:** Multivariate regression analyses of associations between vitamin K intake and SII, SIRI, SIIRI, NLR, white blood cell, neutrophil, monocyte.

SII	Crude model		Model 1		Model 2		Model 3	
Vitamin K intake	β(95%CI)	P	β(95%CI)	P	β(95%CI)	P	β(95%CI)	P
Q1	Reference		Reference		Reference		Reference	
Q2	−20.76 (−33.92, −7.61)	**0.002**	−20.23 (−33.34, −7.13)	**0.003**	−23.05 (−36.66, −9.43)	**0.001**	−19.82 (−33.82, −5.81)	**0.01**
Q3	−21.82 (−33.18, −10.46)	**<0.001**	−22.56 (−33.94, −11.17)	** < 0.001**	−25.97 (−37.90, −14.03)	**<0.0001**	−20.79 (−32.99, −8.59)	**0.001**
Q4	−40.46 (−53.40, −27.53)	**<0.0001**	−42.09 (−54.71, −29.46)	**<0.0001**	−40.19 (−53.41, −26.96)	**<0.0001**	−28.95 (−44.33, −13.56)	**<0.001**
p for trend		**<0.0001**		**<0.0001**		**<0.0001**		**<0.001**
**SIRI**	**Crude model**		**Model 1**		**Model 2**		**Model 3**	
**Vitamin K intake**	**β (95%CI)**	**P**	**β (95%CI)**	**P**	**β (95%CI)**	**P**	**β (95%CI)**	**P**
**Q1**	**Reference**		**Reference**		**Reference**		**Reference**	
Q2	−0.04 (−0.08, 0.01)	0.11	−0.06 (−0.10, −0.02)	**0.01**	−0.06 (−0.10, −0.01)	**0.01**	−0.05 (−0.09, 0.00)	**0.04**
Q3	−0.01 (−0.05, 0.02)	0.50	−0.05 (−0.09, −0.02)	**0.003**	−0.05 (−0.09, −0.02)	**0.005**	−0.05 (−0.09,-0. −01)	**0.01**
Q4	−0.08 (−0.11, −0.04)	**<0.0001**	−0.12 (−0.15, −0.08)	**<0.0001**	-0.09 (−0.13, −0.06)	**<0.0001**	−0.08 (−0.12, −0.03)	**<0.001**
p for trend		**<0.001**		**<0.0001**		**<0.0001**		**0.001**
**SIIRI**	**Crude model**		**Model 1**		**Model 2**		**Model 3**	
**Vitamin K intake**	**β (95%CI)**	**P**	**β (95%CI)**	**P**	**β (95%CI)**	**P**	**β (95%CI)**	**P**
**Q1**	**Reference**		**Reference**		**Reference**		**Reference**	
Q2	−18.42 (−30.47, −6.37)	**0.003**	−20.78 (−32.79, −8.77)	**<0.001**	−20.26 (−32.75, −7.77)	**0.002**	−15.84 (−28.25, −3.43)	**0.01**
Q3	−17.25 (−27.23, −7.27)	**<0.001**	−21.74 (−31.78, −11.71)	**<0.0001**	−21.59 (−31.86, −11.33)	**<0.0001**	−17.29 (−27.47, −7.11)	**0.001**
Q4	−36.88 (−47.03, −26.73)	**<0.0001**	−41.37 (−51.51, −31.22)	**<0.0001**	−35.12 (−45.71, −24.53)	**<0.0001**	−24.7 (−36.79, −12.61)	**<0.001**
p for trend		**<0.0001**		**<0.0001**		**<0.0001**		**<0.001**
**NLR**	**Crude model**		**Model 1**		**Model 2**		**Model 3**	
**Vitamin K intake**	**β (95%CI)**	**P**	**β (95%CI)**	**P**	**β (95%CI)**	**P**	**β (95%CI)**	**P**
**Q1**	**Reference**		**Reference**		**Reference**		**Reference**	
Q2	−0.02 (−0.07,0.03)	0.46	−0.04 (−0.09, 0.01)	0.10	−0.05 (−0.10, 0.00)	**0.04**	−0.05 (−0.10, 0.00)	0.06
Q3	0 (−0.04,0.04)	0.93	−0.04 (−0.08, 0.00)	**0.04**	−0.06 (−0.10, −0.01)	**0.01**	−0.06 (−0.10, −0.02)	**0.01**
Q4	−0.05 (−0.09,0.00)	**0.03**	−0.09 (−0.13, −0.05)	**<0.0001**	−0.09 (−0.13, −0.04)	**<0.001**	−0.08 (−0.14, −0.03)	**0.003**
p for trend		0.05		**<0.0001**		**<0.001**		**0.003**
**WBC**	**Crude model**		**Model 1**		**Model 2**		**Model 3**	
**Vitamin K intake**	**β (95%CI)**	**P**	**β (95%CI)**	**P**	**β (95%CI)**	**P**	**β (95%CI)**	**P**
**Q1**	**Reference**		**Reference**		**Reference**		**Reference**	
Q2	−0.22 (−0.36, −0.08)	**0.002**	−0.2 (−0.33, −0.06)	**0.005**	−0.16 (−0.30, −0.02)	**0.02**	−0.12 (−0.26, 0.01)	0.07
Q3	−0.3 (−0.40, −0.21)	**<0.0001**	−0.26 (−0.36, −0.17)	** < 0.0001**	−0.19 (−0.29, −0.10)	**<0.001**	−0.12 (−0.22, −0.03)	**0.01**
Q4	−0.52 (−0.63, −0.42)	**<0.0001**	−0.49 (−0.59, −0.38)	**<0.0001**	−0.35 (−0.45, −0.24)	**<0.0001**	−0.18 (−0.29, −0.07)	**0.002**
p for trend		** < 0.0001**		**<0.0001**		**<0.0001**		**0.004**
**Neut**	**Crude model**		**Model 1**		**Model 2**		**Model 3**	
**Vitamin K intake**	**β (95%CI)**	**P**	**β (95%CI)**	**P**	**β (95%CI)**	**P**	**β (95%CI)**	**P**
**Q1**	**Reference**		**Reference**		**Reference**		**Reference**	
Q2	−0.18 (−0.27, −0.09)	**<0.0001**	−0.17 (−0.25, −0.08)	**<0.001**	−0.15 (−0.24, −0.07)	**<0.001**	−0.14 (−0.22, −0.06)	**0.001**
Q3	−0.19 (−0.27,-0.12)	**<0.0001**	−0.17 (−0.25, −0.10)	**<0.0001**	−0.15 (−0.22, −0.07)	**<0.001**	−0.1 (−0.17, −0.03)	**0.004**
Q4	−0.36 (−0.44, −0.28)	**<0.0001**	−0.34 (−0.42, −0.26)	**<0.0001**	−0.26 (−0.34, −0.18)	**<0.0001**	−0.15 (−0.23,-0.07)	**<0.001**
p for trend		**<0.0001**		**<0.0001**		**<0.0001**		**0.002**
**MO**	**Crude model**		**Model 1**		**Model 2**		**Model 3**	
**Vitamin K intake**	**β (95%CI)**	**P**	**β (95%CI)**	**P**	**β (95%CI)**	**P**	**β (95%CI)**	**P**
**Q1**	**Reference**		**Reference**		**Reference**		**Reference**	
Q2	−0.01 (−0.02, 0.00)	**0.02**	−0.02 (−0.03, −0.01)	**0.001**	−0.01 (−0.02, 0.00)	**0.01**	−0.01 (−0.02, 0.00)	0.05
Q3	−0.01 (−0.02, 0.00)	0.08	−0.02 (−0.02,-0.01)	**<0.001**	−0.01 (−0.02, 0.00)	**0.02**	−0.01 (−0.02, 0.00)	0.09
Q4	−0.02 (−0.03, −0.01)	**<0.0001**	−0.03 (−0.04, −0.02)	**<0.0001**	−0.02 (−0.03, −0.01)	**<0.0001**	−0.01 (−0.02, 0.00)	**0.01**
p for trend		**<0.0001**		**<0.0001**		**<0.001**		**0.03**

SII, systemic immune-inflammation index; SIRI, systemic inflammation response index; SIIRI, systemic immune-inflammation response index; NLR, neutrophil-to-lymphocyte ratio; WBC, white blood cell; Neut, neutrophil; MO, monocyte. The median (range) of dietary vitamin K intakes for each quartile is as follows: Q1: 25 (0–39.9) mcg/d; Q2: 54.6 (39.9–72.1) mcg/d; Q3: 95.5 (72.1–131.3) mcg/d; Q4: 212.5 (131.3–45067.1) mcg/d. Values in bold indicate statistically significant differences (*P* < 0.05).

After multivariate adjustment including age, gender, race, education, family poverty income ratio, BMI, smoking status, alcohol consumption, vigorous recreational activity, hyperlipidemia, hypertension, and diabetes (Model 3), the highest quartile significantly associated with decreased SII(β = -28.95,95%CI: −44.33 to −13.56, *p* < 0.001) and the p for trend across vitamin K intake categories reaches statistical significance (P trend < 0.001). When vitamin K intake is divided into quartiles, compared to quartile 1, the β and 95%CI in model 3 of quartile 2, quartile 3 and quartile 4 were observed to be −0.05(−0.09, 0.00), −0.05(−0.09, −0.01) and −0.08(−0.12, −0.03) for SIRI (P trend = 0.001). Similarly, the β and 95%CI in model 3 of quartile 2, quartile 3 and quartile 4 were −15.84(−28.25, −3.43), −17.29(−27.47, −7.11) and −24.7(−36.79, −12.61) for SIIRI (P trend < 0.001). Compared to quartile 1, the β and 95%CI in model 3 of quartile 2, quartile 3 and quartile 4 were −0.05(−0.10, 0.00), −0.06(−0.10, −0.02) and −0.08(−0.14, −0.03) for NLR (P trend = 0.003). Compared to quartile 1, the β and 95%CI in model 3 of quartile 2, quartile 3 and quartile 4 were −0.04(−0.06, −0.02), −0.03(−0.05, −0.01) and −0.03(−0.05, −0.01) for RAR (P trend = 0.03). Compared to quartile 1, the β and 95%CI in model 3 of quartile 2, quartile 3 and quartile 4 were −0.12(−0.26, 0.01), −0.12(−0.22, −0.03) and −0.18(−0.29, −0.07) for white blood cell (P trend = 0.004). Compared to quartile 1, the β and 95%CI in model 3 of quartile 2, quartile 3 and quartile 4 were −0.14(−0.22, −0.06), −0.1(−0.17, −0.03) and −0.15(−0.23, −0.07) for neutrophil (P trend = 0.002). Compared to quartile 1, the β and 95%CI in model 3 of quartile 2, quartile 3 and quartile 4 were −0.01(−0.02, 0.00), −0.01(−0.02, 0.00) and −0.01(−0.02, 0.00) for monocyte (P trend = 0.03). Compared to quartile 1, the β and 95%CI in model 3 of quartile 2, quartile 3 and quartile 4 were −0.01(−0.01, 0.00), 0(−0.01, 0.00) and −0.01(−0.02, 0.00) for eosinophil (P trend = 0.05).

However, the inverse association between vitamin K intake and PLR, hs-CRP, lymphocytes, or basophils become insignificant. In the fully adjusted model, the β and 95%CI for PLR in the highest quartile are −2.77(−5.67 to 0.13) and the p for trend is 0.04. In the fully adjusted model, the β and 95%CI for hs-CRP in the highest quartile are −0.28(−0.86, 0.29) and the p for trend is 0.18. In the fully adjusted model, the β and 95%CI for lymphocytes in the highest quartile are −0.01(−0.05, 0.03) and the p for trend is 0.4. In the fully adjusted model, the β and 95%CI for basophils in the highest quartile are 0(−0.01, 0.00) and the p for trend is 0.22.

Surprisingly, a positive relationship between ferritin and vitamin K intake was observed in the crude model, but this association was absent in the other models that are adjusted for variables. Compared to quartile 1, the β and 95%CI in model 3 of quartile 2, quartile 3 and quartile 4 were 8.76(0.86, 16.66), 2.41(−5.06, 9.88) and 2.11(−4.24, 8.46) and the p for trend is 0.98.

### 3.3 The restricted cubic spline

After adjustment for multiple covariates, restricted cubic splines showed downward trends between vitamin K intake and SII, SIRI, SIIRI, NLR, PLR, white blood cell, neutrophil, monocyte. The above inflammatory indicators decreased with the increase of vitamin K intake, but reached a certain extent, beyond which the downward trend was significantly reduced (Supplementary Figure 1).

**FIGURE 2:**
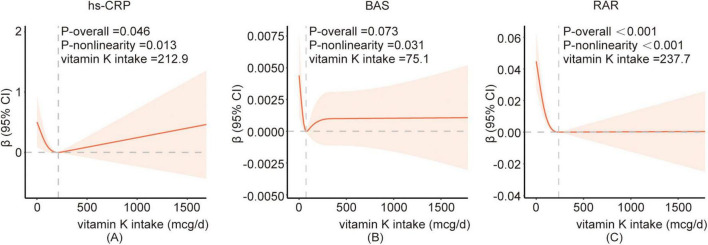
The restricted cubic spline between vitamin K and hs-CRP**(A)**, BAS**(B)**, RAR**(C)**. Adjusted for age, gender, race, family poverty income ratio, educational level, BMI, smoking status, alcohol consumption, vigorous recreational activity, dietary inflammatory index, hyperlipidemia, hypertension, and diabetes. The red curve represents the fitted RCS curve. The light red shaded area indicates the 95% confidence interval for the β estimates. BAS, basophil; hs-CRP, high-sensitivity C- reactive protein; RAR, red blood cell distribution width-to-albumin ratio.

In addition, the data revealed a J-shaped association between vitamin K intake and hs-CRP (Figure 2A) and basophil (Figure 2B), with inflection points at the vitamin K intake value of 212.9 and 75.1 mcg/d, respectively. And piecewise regression was performed according to the inflection point value. The results of segmented regression analysis showed that there was no clear correlation between hs-CRP and vitamin K intake before or after the inflection point. When the vitamin K intake level was lower than the inflection point, the basophil showed a downward trend with the increase of intake, but this association no longer existed when vitamin K intake levels were above 75.1 mcg/d. There was an L-type association between vitamin K intake and RAR (Figure 2C), and when the average daily intake of vitamin K was less than 237.7 mcg/d, RAR showed a significant decreasing trend with the increase of vitamin K, and when it exceeded 237.7 mcg/d, RAR no longer decreased (Table 4; Supplementary Table 2).

**TABLE 4:** Piecewise regressions according to the inflection point value in RAR.

RAR	Crude model		Model 1		Model 2		Model 3	
**Vitamin K levels were below 237.7 mcg/d**								
**Vitamin K intake**	**β (95%CI)**	**P**	**β (95%CI)**	**P**	**β (95%CI)**	**P**	**β (95%CI)**	**P**
Q1: 23.3 (0, 37)	Reference		Reference		Reference		Reference	
Q2: 50.1 (37, 64.2)	−0.05 (−0.08, −0.03)	**<0.0001**	−0.05 (−0.07, −0.02)	**<0.001**	−0.04 (−0.06, −0.02)	**0.001**	−0.03 (−0.05, −0.01)	**0.001**
Q3: 82.7 (64.2, 107.1)	−0.07 (−0.10, −0.04)	**<0.0001**	−0.07 (−0.10, −0.04)	**<0.0001**	−0.06 (−0.09, −0.03)	**<0.0001**	−0.04 (−0.06, −0.02)	**<0.001**
Q4: 146.2 (107.1, 237.7)	−0.09 (−0.12, −0.06)	**<0.0001**	−0.09 (−0.11, −0.06)	**<0.0001**	−0.07 (−0.10, −0.05)	**<0.0001**	−0.04 (−0.06, −0.02)	**<0.001**
p for trend		** < 0.0001**		**<0.0001**		**<0.0001**		**0.001**
**Vitamin K levels were above 237.7 mcg/d**								
**Vitamin K intake**	**β (95%CI)**	**P**	**β (95%CI)**	**P**	**β (95%CI)**	**P**	**β (95%CI)**	**P**
Q1: 257.4 (237.8, 281.7)	Reference		Reference		Reference		Reference	
Q2: 314.4 (281.7, 357.1)	−0.02 (−0.07, 0.04)	0.56	−0.02 (−0.08, 0.03)	0.42	−0.01 (−0.06, 0.03)	0.55	0 (−0.05, 0.04)	0.91
Q3: 421.2 (357.1, 536.4)	−0.06 (−0.11, 0.00)	**0.03**	−0.07 (−0.12, −0.02)	**0.01**	−0.05 (−0.10, 0.00)	0.05	−0.05 (−0.09, −0.01)	**0.02**
Q4: 763.7 (536.4, 45067.1)	−0.04 (−0.10, 0.02)	0.15	−0.04 (−0.09, 0.02)	0.16	−0.06 (−0.11, 0.00)	**0.04**	−0.02 (−0.07, 0.03)	0.51
p for trend		0.06		0.05		**0.01**		0.16

RAR, red blood cell distribution width-to-albumin ratio. Values in bold indicate statistically significant differences (*P* < 0.05).

### 3.4 Subgroup analysis

It was found that the reduction of various inflammatory indicators was not consistently associated with increased vitamin K intake levels (Supplementary Table 3) in certain subgroups and several results were not statistically significant.

The data revealed a stronger negative correlation between vitamin K intake and SII, SIIRI, NLR, PLR in people over 60 years of age, although the interaction test was not significant. In men, there was a more significant negative association between vitamin K intake and SII, SIRI, SIIRI, NLR, white blood cell, neutrophil, eosinophil, basophil. In women, only PLR was negatively correlated with vitamin K intake. However, the interaction tests were not distinct on age, sex, ethnicity, BMI, hyperlipidemia, diabetes, and hypertension on the negative correlation between SII, NLR, PLR, lymphocyte, and vitamin K. In hyperlipidemia patients, only SII, SIIRI, PLR, and eosinophil were negatively correlated with vitamin K intake, and in DM patients, only basophil was negatively correlated with vitamin K intake (p for interaction = 0.005). In hypertensive patients, only eosinophil was negatively correlated with vitamin K intake, which was statistically significant (p for interaction = 0.063). The interaction analysis revealed that diabetes exhibited a correlation with the relationship between vitamin K intake and ferritin, basophil, while hyperlipidemia was associated with the relationship between vitamin K intake and hs-CRP, and hypertension demonstrated a correlation with the relationship between vitamin K intake and ferritin.

## 4 Discussion

In the present study, the association between vitamin K intake and various cellular immune inflammation markers was explored within a large and random sample of American adults. To the best of current knowledge, this is the first attempt to assess the relationship between vitamin K intake and blood inflammation using the NHANES database. The results manifested that vitamin K intake had a negative relationship with SII, SIRI, SIIRI, NLR, leukocytes, neutrophils, and monocytes.

SII, SIRI and SIIRI, as novel biomarkers that integrate various leukocyte subsets, were recently devised to assess the balance between inflammation and immune response, which have great potential in clinical settings (34, 35). SII and SIRI were higher in patients diagnosed with acute coronary syndrome (ACS) compared to those with stable coronary artery disease (CAD) (18). And elevated SII or SIIRI was associated with poor cardiovascular prognosis in patients with CAD (35, 36). NLR is also an emerging marker in inflammatory diseases (37). NLR is independently correlated with CAD severity and 3-year outcomes (38) and has a better ability to predict in-hospital death after NSTEMI (39). A significant correlation between NLR and the survival rate was observed in Amyotrophic lateral sclerosis (ALS) patients, and increased NLR at diagnosis was associated with poorer survival outcomes. NLR was a risk factor for the low survival rate of ALS patients and a faster rate of disease progression was associated with a higher NLR value (40–42). RAR, another easy, economical predictor, has been shown to be associated with clinical outcomes in patients with diabetic retinopathy (43), respiratory distress syndrome (32), and burns (44).

Vitamin K exists in various isoforms, primarily distinguished by two structures: phylloquinone (vitamin K1) and menaquinones (vitamin K2), both of which serve as cofactors for the γ-glutamyl carboxylase enzyme. The menaquinone (MK) family is further divided into multiple isoforms, designated as MK-n, where n stands for the number of isoprene residues. Vitamin K2 accounts for 10–25% of total dietary vitamin K intake (45). Vitamin K1 exhibits a half-life of approximately 3 h, and only 10–15% of it is absorbed by the body. In contrast, MK-7 has a half-life of around 72 h and can persist in circulation for up to 144 h, with more efficient absorption (46). While vitamin K1 is preferentially retained in the liver to assist in the carboxylation of clotting factors, vitamin K2, particularly the long-chain derivatives, is redistributed into circulation and becomes available for extrahepatic tissues, such as bone and blood vessels (47). Vitamin K2 can also act as a mitochondrial electron carrier, effectively improving mitochondrial dysfunction and enhancing ATP production (48). In 2010, Yusuke Ohsaki with his colleagues have shown that vitamin K can suppress the lipopolysaccharide-induced inflammation via the inhibition of the activation of nuclear factor kappa B(NF-κB) through the repression of IKKα/β phosphorylation (49). In this study, all vitamin K forms (K1, MK3, MK4 and MK7) were found to suppress Interleukin-6 (IL-6) mRNA expression. NF-κB is one of the most important regulators of proinflammatory gene expression, and the activation of NF-κB promotes the production of inflammatory factors such as IL-1, IL-6, IL-8, and TNF-α (50). Vitamin K has been shown to inhibit the NF-κB signaling pathway, thereby reducing the production of inflammatory factors and potentially preventing vascular calcification (51). The carboxylation of Matrix Gla Protein (MGP) by vitamin K results in its high-affinity calcium binding, which in turn inhibits vascular calcification (52). Both Vitamin K1 and vitamin K2 are beneficial for neurocognitive functions via the regulation of sphingolipid metabolism (53, 54). Studies have shown that different forms of vitamin K2 (MK-4 and MK-7) can suppress the production of proinflammatory cytokines such as TNF-α, IL-1β, and IL-6, thereby reducing neuroinflammation in different types of glial cells (55). Another investigation showed that not only MK but also vitamin K1 may play protective roles in endothelial function, cell senescence and vascular inflammation (56). The vasoprotective effect of vitamin K1 could be attributed either to vitamin K1 itself or to the action of MK that is formed from vitamin K1. The inhibitory effect of vitamin K1 and MK on inflammatory markers was consistent with the effects on the activation of the NF-κB pathway. High levels of the reduced form of vitamin K can reduce ferroptosis by inhibiting lipid peroxidation and help protect cells from oxidative stress by increasing the expression of antioxidant enzymes (57). This provides a possible explanation for the positive correlation between vitamin K and ferritin in the crude model. The role of vitamin K on gastrointestinal health has also attracted more and more attention, as it can alleviate intestinal inflammation, regulate microbial metabolites, and improve intestinal flora (58).

Despite a growing body of research investigating the relationship between vitamin K intake and inflammatory markers, the findings have been inconsistent. Kristensen M et al conducted a double-blind, randomized crossover study involving 31 postmenopausal women and observed that, although phylloquinone supplementation resulted in a reduction in IL-6 levels (from 1.99 to 1.63 pg/mL) but did not reach statistical significance (59). Moreover, although the mean CRP concentration was significantly lower after the phylloquinone intervention when considering all participants, this significance was lost when applying a cutoff value of 10 mg/l. In contrast, an inverse association between vitamin K status and IL-6 was observed in one community-based cohort by measuring plasma phylloquinone and phylloquinone intake (60). A cross-sectional and longitudinal study with 568 elderly subjects at high cardiovascular risk found that an increased intake of phylloquinone was associated with significant improvements in inflammatory markers (such as IL-6 and TNF) (61), thereby supporting the protective effect of vitamin K against low-grade chronic inflammatory diseases. However, in another study involving 662 participants, Shea MK et al found that serum phylloquinone levels exhibited a negative correlation with IL-6 and CRP, whereas dietary phylloquinone intake did not show a significant association with these inflammatory markers (62). Furthermore, Nakajima S et al observed a significant correlation between vitamin K deficiency and clinical disease activity in patients with Crohn’s disease (63). The discrepancies in these study findings may stem from differences in the health status of the study subjects, sample size, measurement methods, and the degree of control for confounding factors. Overall, these studies highlight the complexity of the relationship between vitamin K and inflammatory markers in different populations. The present study, drawing data from the NHANES database, revealed an inverse association between vitamin K intake and several inflammatory biomarkers, albeit not all. This correlation suggests that higher vitamin K intake may be linked to a reduced inflammatory status, potentially implicating vitamin K in the regulation of inflammation. Moreover, these findings indicate that vitamin K intake could be associated with a lower risk of chronic inflammatory diseases and mortality.

Based on bilateral knee radiographs and MRI scans obtained at baseline and 30-month follow-up, as well as plasma phylloquinone concentrations obtained at baseline, Misra D et al found that subclinical vitamin K deficiency was associated with an increased risk of developing knee osteoarthritis (64). Later, Shea M K et al also found out that participants with very low plasma phylloquinone were more likely to have worsening of articular cartilage damage and meniscal damage at 3 years of follow-up (65). Lower vitamin K level was associated with lower ventilatory capacity, and with higher risk of lung disease/symptoms (66). Severe vitamin K deficiency in hospitalized patients with COVID-19 infection was related to poor outcome (67). Baseline vitamin K deficiency may be associated with disease progression or increased mortality, but there are also studies suggesting that inflammation-induced vitamin K depletion during infection may be the most important cause of vitamin K deficiency in COVID-19 patients (68).

The current recommended daily intake of vitamin K ranges from 55 to 120 μg/day (69), which can be obtained from healthy diets rich in fruits and vegetables and dietary supplements. But our data revealed that 37.78% of people still consumed less than the minimum recommended dose of vitamin K. Second, it is still unclear whether this dosage supply meets physiological needs unrelated to blood clotting, such as maintaining healthy bones, brain, and blood vessels. In one NHANES data analysis, high intakes of dietary vitamin K may reduce the risk of HPV infection. From the non-linear relationship between dietary vitamin K and HPV infection, the authors recommended a dietary vitamin K intake of more than 14.03 μg (70). The data in the present study also showed that when the vitamin K content exceeded a certain level, certain inflammatory indicators tended to stabilize, while other inflammatory indicators may increase accordingly, such as hs-CRP and BAS. The analysis revealed a non-linear association between vitamin K intake and hs-CRP, basophil count, and RAR. The inflection points were observed at 212.9 and 75.1 mcg/d for hs-CRP and basophil count, respectively, while an L-shaped relationship was found with RAR at an inflection point of 237.7 mcg/d of vitamin K intake. It suggested that a vitamin K intake of approximately 237.7 mcg/d may lead to stabilization of inflammatory markers, indicating a potential reference value for clinical supplementation levels. However, further research is required. Whether excessive vitamin K increases the risk of chronic inflammation or whether inflammation causes high vitamin K consumption still needs to be further confirmed in clinical trials.

In the study, borderline *p*-values for trends were observed for certain outcomes. In model 3, vitamin K intake in the third and fourth quartiles exhibited a significant or nearly significant negative association with PLR. Trend analysis disclosed a significant inverse relationship between vitamin K intake and PLR (p for trend = 0.04), indicating that PLR might decline as vitamin K intake rises. Nevertheless, this relationship was not evident in the other models. The restricted cubic spline plot demonstrated that the decline in PLR gradually lessened with increasing vitamin K intake, suggesting a potential threshold effect of vitamin K intake. Specifically, its influence on PLR seems to reach a stable state after a certain intake level is attained. In both the crude model and Model 1, a significant negative trend relationship was observed between vitamin K intake and hs-CRP levels. In Model 2, this trend relationship persisted, yet it did not reach statistical significance. In Model 3, this trend relationship was no longer significant. This indicates that after further adjustment for variables, the relationship between vitamin K intake and hs-CRP level became insignificant, suggesting that it may be influenced by other variables. Although hs-CRP is a widely utilized marker, the effect of vitamin K might be manifested in other more specific inflammatory pathways or markers, such as IL-6, TNF-α, or other cytokines. To fully comprehend the anti-inflammatory effect of vitamin K, more potential factors and mechanisms still need to be explored.

Compared to previous studies based on the NHANES database or population studies, this research exhibited certain differences. It encompassed a longer time frame and was not limited to middle-aged and elderly individuals. This resulted in a larger sample size, a broader age range, and a more diverse racial composition, thereby enhancing the representativeness and generalizability of the findings. The use of the AMPM method for dietary assessment enhanced reliability over traditional Food Frequency Questionnaire. Unlike other studies that primarily focused on inflammatory markers such as IL-6 and TNF, this research involved more readily available indicators and incorporated new inflammatory calculation indices, such as SIIRI. This study did not confine its investigation to specific conditions or diseases but rather broadly examined the potential connections between vitamin K intake and various inflammatory factors. The study suggested a potential dose-response relationship between vitamin K and inflammation, providing a new theoretical perspective. Results indicated a negative correlation between dietary vitamin K and certain inflammatory factors, highlighting the complexity of their relationship and the need for further investigation into vitamin K’s role across different inflammation indicators.

The study involved 14 inflammatory biomarkers, expanding the understanding of the anti-inflammatory and immunomodulatory effects of vitamin K. It must be acknowledged that there are some limitations in this study. This study relied on 24-h dietary recall interviews and did not accurately reflect long-term vitamin K intake in participants. It is acknowledged that reliance on a single day of assessment may not fully capture the habitual variation in vitamin K consumption. Meanwhile, recall bias, which is caused by factors such as memory bias, social expectation bias, and dietary diurnal variability, may result in an underestimation or overestimation of the intake of certain foods or nutrients. Given the limitations of dietary assessment methods in cross-sectional studies, there is a recognized need for more robust approaches. Future work with longer-term dietary assessments is warranted. Vitamin K is not measured in all types of foods, in other words, the food composition list may be incomplete, especially vitamin K2. Vitamin K2 exhibits a longer half-life in circulation and superior absorption compared to vitamin K1, with MK-7 being the most bioavailable among the various MKs (47). There is growing evidence suggesting that the role of vitamin K2 differs from that of vitamin K1 (71). While vitamin K1 primarily supports coagulation function, vitamin K2 plays a more significant role in maintaining bone health and cardiovascular wellbeing. However, the data in this study did not differentiate between vitamin K1 and vitamin K2. There are individual differences in vitamin K absorption, and their intake does not reflect actual vitamin K status. Vitamin K1 is the plant-based phylloquinone, which is found in green leafy vegetables and vegetable oils. Its intake is intrinsically combined with health-conscious dietary patterns and lifestyle factors, which are also known to exert anti-inflammatory effects. Although the markers in this study have the potential for clinical applications, their accuracy and reliability may require extensive data for further validation. However, this study did not investigate additional biomarkers such as tumor necrosis factor and interleukin. The association between vitamin K and tumor necrosis factor and other indicators could be further explored in future clinical studies. A cross-sectional study design is not possible to make causal inferences about the relationship between vitamin K and blood inflammatory markers. Inflammation could lead to changes in dietary behaviors that result in lower vitamin K intake or could directly affect vitamin K metabolism, potentially lowering vitamin K levels in the blood. The study design does not allow us to determine whether the observed association is due to vitamin K influencing inflammation or inflammation influencing vitamin K levels. This bidirectional relationship complicates the interpretation of the findings. Future research should consider longitudinal studies or randomized controlled trials to better address this issue. The study did not consider the synergistic effects or interactions of vitamin K with other vitamins (e.g., A, B, C, D) or drugs (e.g., folic acid).

## 5 Conclusion

In conclusion, the results indicated a negative association between vitamin K intake and SII, SIRI, SIIRI, NLR, leukocytes, neutrophils, and monocytes. This suggested a potential link between vitamin K and inflammation regulation. However, given the cross-sectional nature of the study, causality cannot be established. Vitamin K may play a role in the development of chronic inflammation. Further prospective studies are needed to explore the potential role of vitamin K supplementation in modifying inflammation.

## Data Availability

The original contributions presented in the study are included in the article/Supplementary material, further inquiries can be directed to the corresponding author.

## Footnotes

1
https://www.cdc.gov/nchs/nhanes/index.htm

2
https://www.cdc.gov/nchs/nhanes/irba98.htm
