# Supplementary material for: Associations between vitamin K and systemic immune and inflammation biomarkers: a population-based study from the NHANES (2007–2020)
Source: Front Nutr. 2025 Jul 11;12:1625209. doi: 10.3389/fnut.2025.1625209 (PMC12289624; doi:10.3389/fnut.2025.1625209)
Supplement: Supplementary file 1 [file Table_1.docx]

**Supplementary Table 1 Multivariate regression analyses of associations between Vitamin K intake and other inflammatory biomarkers**

| PLR | Crude model | | Model 1 |  | Model 2 |  | Model 3 |  |
| --- | --- | --- | --- | --- | --- | --- | --- | --- |
| Vitamin K intake | β(95%CI) | P | β(95%CI) | P | β(95%CI) | P | β(95%CI) | P |
| Q1 | reference |  | reference |  | reference |  | reference |  |
| Q2 | -0.46(-2.48,1.57) | 0.65 | -0.53(-2.53, 1.46) | 0.60 | -1.32(-3.38, 0.75) | 0.21 | -1(-3.22, 1.21) | 0.37 |
| Q3 | -0.09(-2.11,1.93) | 0.93 | -0.59(-2.61, 1.44) | 0.57 | -2.12(-4.33, 0.08) | 0.06 | -2.48(-4.86,-0.11) | **0.04** |
| Q4 | 0.46(-1.84,2.75) | 0.69 | -0.22(-2.48, 2.03) | 0.85 | -1.98(-4.44, 0.48) | 0.11 | -2.77(-5.67, 0.13) | 0.06 |
| p for trend | | 0.6 |  | 0.87 |  | 0.1 |  | **0.04** |
| RAR | Crude model | | Model 1 |  | Model 2 |  | Model 3 |  |
| Vitamin K intake | β(95%CI) | P | β(95%CI) | P | β(95%CI) | P | β(95%CI) | P |
| Q1 | reference |  | reference |  | reference |  | reference |  |
| Q2 | -0.06(-0.08,-0.03) | **<0.0001** | -0.05(-0.08,-0.03) | **<0.0001** | -0.04(-0.06,-0.01) | **0.002** | -0.04(-0.06,-0.02) | **<0.0001** |
| Q3 | -0.07(-0.09,-0.04) | **<0.0001** | -0.07(-0.09,-0.04) | **<0.0001** | -0.04(-0.07,-0.01) | **0.01** | -0.03(-0.05,-0.01) | **0.01** |
| Q4 | -0.09(-0.12,-0.06) | **<0.0001** | -0.1(-0.13,-0.07) | **<0.0001** | -0.06(-0.09,-0.03) | **<0.0001** | -0.03(-0.05,-0.01) | **0.01** |
| p for trend | | **<0.0001** |  | **<0.0001** |  | **<0.001** |  | **0.03** |
| ferritin | Crude model | | Model 1 |  | Model 2 |  | Model 3 |  |
| Vitamin K intake | β(95%CI) | P | β(95%CI) | P | β(95%CI) | P | β(95%CI) | P |
| Q1 | reference |  | reference |  | reference |  | reference |  |
| Q2 | 13.98( 6.37,21.60) | **<0.001** | 2.07(-4.81, 8.95) | 0.55 | 3.04(-4.41,10.49) | 0.42 | 8.76(0.86, 16.66) | 0.03 |
| Q3 | 22.86(14.84,30.88) | **<0.0001** | 1.17(-6.22, 8.55) | 0.75 | 1.43(-6.17, 9.03) | 0.71 | 2.41(-5.06, 9.88) | 0.52 |
| Q4 | 21.24(12.85,29.62) | **<0.0001** | -2.24(-8.30, 3.82) | 0.46 | -3.09(-9.62, 3.45) | 0.35 | 2.11(-4.24, 8.46) | 0.51 |
| p for trend | | **<0.0001** |  | 0.37 |  | 0.24 |  | 0.98 |
| hs-CRP | Crude model | | Model 1 |  | Model 2 |  | Model 3 |  |
| Vitamin K intake | β(95%CI) | P | β(95%CI) | P | β(95%CI) | P | β(95%CI) | P |
| Q1 | reference |  | reference |  | reference |  | reference |  |
| Q2 | -0.6(-1.30, 0.10) | 0.09 | -0.55(-1.26, 0.16) | 0.12 | -0.3(-1.02, 0.42) | 0.40 | -0.15(-0.73, 0.43) | 0.60 |
| Q3 | -1.02(-1.67,-0.37) | **0.003** | -0.98(-1.65,-0.31) | **0.005** | -0.73(-1.42,-0.05) | **0.04** | -0.49(-1.04, 0.06) | 0.08 |
| Q4 | -1.21(-1.99,-0.44) | **0.003** | -1.17(-1.96,-0.39) | **0.004** | -0.74(-1.63, 0.15) | 0.10 | -0.28(-0.86, 0.29) | 0.32 |
| p for trend | | **<0.001** |  | **0.001** |  | 0.06 |  | 0.18 |
| Lym | Crude model | | Model 1 |  | Model 2 |  | Model 3 |  |
| Vitamin K intake | β(95%CI) | P | β(95%CI) | P | β(95%CI) | P | β(95%CI) | P |
| Q1 | reference |  | reference |  | reference |  | reference |  |
| Q2 | -0.02(-0.10, 0.05) | 0.54 | 0(-0.08, 0.07) | 0.90 | 0.02(-0.06, 0.09) | 0.68 | 0.03(-0.06, 0.12) | 0.49 |
| Q3 | -0.1(-0.13,-0.06) | **<0.0001** | -0.07(-0.10,-0.03) | **<0.001** | -0.03(-0.07, 0.01) | 0.11 | -0.01(-0.05, 0.03) | 0.68 |
| Q4 | -0.12(-0.16,-0.08) | **<0.0001** | -0.09(-0.13,-0.05) | **<0.0001** | -0.05(-0.09,-0.01) | **0.01** | -0.01(-0.05, 0.03) | 0.66 |
| p for trend | | **<0.0001** |  | **<0.0001** |  | **0.02** |  | 0.4 |
| EOS | Crude model | | Model 1 |  | Model 2 |  | Model 3 |  |
| Vitamin K intake | β(95%CI) | P | β(95%CI) | P | β(95%CI) | P | β(95%CI) | P |
| Q1 | reference |  | reference |  | reference |  | reference |  |
| Q2 | 0(-0.01, 0.00) | 0.17 | -0.01(-0.01, 0.00) | **0.04** | -0.01(-0.01, 0.00) | 0.15 | -0.01(-0.01, 0.00) | 0.18 |
| Q3 | 0(-0.01, 0.00) | 0.48 | -0.01(-0.01, 0.00) | 0.06 | 0(-0.01, 0.00) | 0.33 | 0(-0.01, 0.00) | 0.39 |
| Q4 | -0.01(-0.02,-0.01) | **<0.001** | -0.02(-0.02,-0.01) | **<0.0001** | -0.01(-0.02,-0.01) | **<0.001** | -0.01(-0.02, 0.00) | **0.03** |
| p for trend | | **<0.001** |  | **<0.0001** |  | **0.001** |  | 0.05 |
| BAS | Crude model | | Model 1 |  | Model 2 |  | Model 3 |  |
| Vitamin K intake | β(95%CI) | P | β(95%CI) | P | β(95%CI) | P | β(95%CI) | P |
| Q1 | reference |  | reference |  | reference |  | reference |  |
| Q2 | 0(-0.01,0.00) | **<0.001** | 0(-0.01,0.00) | **<0.001** | 0(-0.01,0.00) | **0.002** | 0(-0.01,0.00) | **0.03** |
| Q3 | -0.01(-0.01,0.00) | **<0.0001** | -0.01(-0.01,0.00) | **<0.0001** | 0(-0.01,0.00) | **<0.001** | 0(-0.01,0.00) | 0.06 |
| Q4 | -0.01(-0.01,0.00) | **<0.0001** | -0.01(-0.01,0.00) | **<0.0001** | 0(-0.01,0.00) | **<0.0001** | 0(-0.01,0.00) | 0.15 |
| p for trend | | **<0.0001** |  | **<0.0001** |  | **<0.001** |  | 0.22 |

Abbreviations: PLR, platelet-to-lymphocyte ratio; RAR, red blood cell distribution width-to-albumin ratio; hs-CRP, high-sensitivity C- reactive protein; Lym, lymphocyte; EOS, eosinophil; BAS, basophil.

The median (range) of dietary vitamin K intakes for each quartile is as follows: Q1: 25 (0–39.9) mcg/d; Q2: 54.6(39.9-72.1) mcg/d; Q3: 95.5(72.1-131.3) mcg/d; Q4: 212.5(131.3-45067.1) mcg/d.
