# Supplementary material for: Associations between vitamin K and systemic immune and inflammation biomarkers: a population-based study from the NHANES (2007–2020)
Source: Front Nutr. 2025 Jul 11;12:1625209. doi: 10.3389/fnut.2025.1625209 (PMC12289624; doi:10.3389/fnut.2025.1625209)
Supplement: Supplementary file 2 [file Table_2.docx]

**Supplementary Table 2 Piecewise regressions according to the inflection point value in BAS and hs-CRP**

| **vitamin K levels were below 75.1 mcg/d** | | | | | | | | |
| --- | --- | --- | --- | --- | --- | --- | --- | --- |
| BAS | Crude model |  | Model 1 |  | Model 2 |  | Model 3 |  |
| Vitamin K intake | β（95%CI） | P | β（95%CI） | P | β（95%CI） | P | β（95%CI） | P |
| Q1: 16.7 (0,25.5) | reference |  | reference |  | reference |  | reference |  |
| Q2: 33.4 (25.5,41) | 0( 0.00,0.00) | 0.48 | 0(-0.01,0.00) | 0.40 | 0( 0.00, 0.00) | 0.95 | 0( 0.00, 0.00) | 0.71 |
| Q3: 48.8 (41,56.4) | 0(-0.01,0.00) | **0.01** | 0(-0.01,0.00) | **0.01** | 0(-0.01, 0.00) | 0.05 | 0(-0.01, 0.00) | 0.32 |
| Q4: 65 (56.4,75) | -0.01(-0.01,0.00) | **<0.001** | -0.01(-0.01,0.00) | **<0.001** | 0(-0.01, 0.00) | **0.003** | 0(-0.01, 0.00) | **0.02** |
| p for trend |  | **<0.001** |  | **<0.0001** |  | **<0.001** |  | **0.01** |
| **vitamin K levels were above 75.1 mcg/d** | | | | | | | | |
| BAS | Crude model |  | Model 1 |  | Model 2 |  | Model 3 |  |
| Vitamin K intake | β（95%CI） | P | β（95%CI） | P | β（95%CI） | P | β（95%CI） | P |
| Q1: 86 (75.1,98.7) | reference |  | reference |  | reference |  | reference |  |
| Q2: 114.7 (98.7,135.5) | 0( 0.00,0.00) | 0.52 | 0( 0.00,0.00) | 0.55 | 0( 0.00, 0.00) | 0.49 | 0( 0.00, 0.00) | 0.87 |
| Q3: 166.6 (135.5,216.8) | 0( 0.00,0.00) | 0.71 | 0( 0.00,0.00) | 0.71 | 0( 0.00, 0.00) | 0.82 | 0( 0.00, 0.00) | 0.96 |
| Q4: 327.15 (216.8,45067.1) | 0(-0.01,0.00) | 0.06 | 0(-0.01,0.00) | 0.05 | 0(-0.01, 0.00) | 0.21 | 0( 0.00, 0.01) | 0.42 |
| p for trend |  | 0.09 |  | 0.08 |  | 0.32 |  | 0.43 |
| **vitamin K levels were below 212.9 mcg/d** | | | | | | | | |
| hs-CRP | Crude model |  | Model 1 |  | Model 2 |  | Model 3 |  |
| Vitamin K intake | β（95%CI） | P | β（95%CI） | P | β（95%CI） | P | β（95%CI） | P |
| Q1: 23 (0,36.4) | reference |  | reference |  | reference |  | reference |  |
| Q2: 49.2 (36.4,62.7) | -0.48(-1.11, 0.15) | 0.14 | -0.43(-1.07, 0.20) | 0.18 | -0.34(-1.11, 0.42) | 0.37 | 0.04(-0.49, 0.56) | 0.88 |
| Q3: 80.1 (62.7,102.8) | -0.82(-1.54,-0.10) | **0.03** | -0.79(-1.53,-0.05) | **0.04** | -0.56(-1.38, 0.25) | 0.17 | -0.08(-0.58, 0.42) | 0.75 |
| Q4: 138.4 (102.8,212.9) | -1.21(-1.79,-0.63) | **<0.001** | -1.18(-1.77,-0.59) | **<0.001** | -0.94(-1.57,-0.31) | **0.004** | -0.27(-0.69, 0.15) | 0.20 |
| p for trend |  | **<0.0001** |  | **<0.0001** |  | **0.002** |  | 0.14 |
| **vitamin K levels were above 212.9 mcg/d** | | | | | | | | |
| hs-CRP | Crude model |  | Model 1 |  | Model 2 |  | Model 3 |  |
| Vitamin K intake | β（95%CI） | P | β（95%CI） | P | β（95%CI） | P | β（95%CI） | P |
| Q1: 230.9 (213,252.75) | reference |  | reference |  | reference |  | reference |  |
| Q2: 281.5 (252.75,321.5) | 1.02(-1.33,3.37) | 0.39 | 0.96(-1.41, 3.34) | 0.42 | 1.18(-0.91, 3.27) | 0.26 | 1.22(-0.39, 2.83) | 0.13 |
| Q3: 378.65 (321.5,480.1) | -0.06(-0.96,0.84) | 0.89 | -0.35(-1.29, 0.58) | 0.45 | 0.02(-1.01, 1.04) | 0.97 | -0.22(-1.73, 1.28) | 0.76 |
| Q4: 697.5 (480.1,45067.1) | -0.16(-1.10,0.77) | 0.73 | -0.37(-1.29, 0.55) | 0.42 | -0.21(-1.08, 0.67) | 0.63 | -0.02(-1.23, 1.18) | 0.97 |
| p for trend |  | 0.39 |  | 0.2 |  | 0.31 |  | 0.57 |

Abbreviations: BAS, basophil; hs-CRP, high-sensitivity C- reactive protein.
