# Supplementary material for: Associations between vitamin K and systemic immune and inflammation biomarkers: a population-based study from the NHANES (2007–2020)
Source: Front Nutr. 2025 Jul 11;12:1625209. doi: 10.3389/fnut.2025.1625209 (PMC12289624; doi:10.3389/fnut.2025.1625209)
Supplement: Supplementary file 3 [file Table_3.docx]

**Supplementary Table 3** Subgroup analysis for the association between inflammatory biomarkers and vitamin K intake

**Summary Table of Subgroup Analysis Results (Marked for P<0.05)**

| Character | **SII** | **SIRI** | **SIIRI** | **NLR** | **PLR** | **RAR** | **ferritin** | **hs-CRP** | **WBC** | **Neut** | **Lym** | **MO** | **EOS** | **BAS** |
| --- | --- | --- | --- | --- | --- | --- | --- | --- | --- | --- | --- | --- | --- | --- |
| Age [20,45) |  |  |  |  |  |  |  |  |  |  |  |  | 0.025 |  |
| Age [45,60) |  |  |  |  |  |  |  |  |  |  |  |  |  |  |
| Age [60,) | 0.025 |  | 0.021 | 0.027 | 0.006 |  | 0.022 |  |  |  |  |  |  |  |
| Female |  |  |  |  | 0.048 |  |  |  |  |  |  |  |  |  |
| Male | 0.005 | 0.041 | 0.031 | 0.018 |  |  |  |  | 0.016 | 0.019 |  |  | 0.029 | 0.044 |
| Non-Hispanic White | 0.003 |  | 0.019 | 0.014 | 0.015 |  |  | 0.008 | 0.038 | 0.037 |  |  | 0.015 |  |
| Non-Hispanic Black |  |  |  |  |  |  |  |  |  |  |  |  |  |  |
| Mexican American |  |  |  |  |  |  |  |  |  |  |  |  |  |  |
| Other Hispanic | 0.025 |  |  | 0.015 | <0.0001 | <0.0001 |  |  |  |  | <0.0001 |  |  | 0.006 |
| Other Race | 0.036 | <0.0001 | <0.001 | 0.007 |  |  |  |  |  | 0.012 |  | <0.001 | 0.006 |  |
| BMI group |  |  |  |  |  |  |  |  |  |  |  |  |  |  |
| underweight |  |  |  |  |  |  |  |  | 0.039 |  | 0.032 |  |  | 0.048 |
| normal |  |  | 0.03 |  |  |  |  |  |  |  |  |  | 0.02 |  |
| overweight | 0.004 | 0.015 | 0.041 | <0.001 |  |  |  |  |  | 0.024 |  |  | 0.002 | 0.036 |
| obese |  |  |  |  | 0.027 |  |  |  |  |  |  |  |  |  |
| Hyperlipidemia | 0.017 |  | 0.032 |  | 0.004 |  |  |  |  |  |  |  | 0.01 |  |
| No- Hyperlipidemia |  | 0.041 | 0.048 |  |  |  |  | 0.041 |  |  |  |  |  |  |
| Diabetes |  |  |  |  |  |  |  |  |  |  |  |  |  | 0.003 |
| No- Diabetes | 0.01 | 0.042 | 0.019 | 0.016 | 0.017 |  |  |  |  |  |  |  | 0.018 |  |
| Hypertension |  |  |  |  |  |  |  |  |  |  |  |  | 0.005 |  |
| No- Hypertension | 0.024 | 0.041 | 0.031 | 0.035 | 0.015 |  |  |  |  |  |  |  | 0.017 |  |

To facilitate review, all values with P<0.05 in the subgroup analyses are highlighted in the summary table of subgroup analyses.

Specifically, P for interaction<0.05 is indicated with an orange background.

**Supplementary Table 3** Subgroup analysis for the association between inflammatory biomarkers and vitamin K intake

| **Supplementary Table S1 Subgroup analysis for the association between SII and vitamin K intake.** | | | |
| --- | --- | --- | --- |
| character | β(95% CI) | p | p for interaction |
| Age group |  |  | 0.938 |
| [20,45) | -0.019(-0.052, 0.015) | 0.266 |  |
| [45,60) | -0.014(-0.031, 0.002) | 0.092 |  |
| [60,) | -0.016(-0.029, -0.002) | 0.025 |  |
| Gender |  |  | 0.147 |
| Female | -0.007(-0.018, 0.003) | 0.162 |  |
| Male | -0.031(-0.052, -0.009) | 0.005 |  |
| Race |  |  | 0.086 |
| Non-Hispanic White | -0.026( -0.043, -0.009) | 0.003 |  |
| Non-Hispanic Black | 0.019( -0.050, 0.087) | 0.587 |  |
| Mexican American | 0.064( -0.062, 0.189) | 0.315 |  |
| Other Hispanic | -0.006( -0.011, -0.001) | 0.025 |  |
| Other Race | -0.074( -0.143, -0.005) | 0.036 |  |
| BMI group |  |  | 0.272 |
| underweight | 0.113( -0.272, 0.498) | 0.559 |  |
| normal | -0.011(-0.022, 0.000) | 0.05 |  |
| overweight | -0.068(-0.113, -0.022) | 0.004 |  |
| obese | -0.018(-0.047, 0.012) | 0.237 |  |
| Hyperlipidemia |  |  | 0.678 |
| yes | -0.014(-0.026, -0.003) | 0.017 |  |
| no | -0.034(-0.071, 0.004) | 0.08 |  |
| Diabetes |  |  | 0.936 |
| yes | -0.006(-0.046, 0.034) | 0.755 |  |
| no | -0.017(-0.029, -0.004) | 0.01 |  |
| Hypertension |  |  | 0.256 |
| yes | -0.031(-0.067, 0.004) | 0.082 |  |
| no | -0.012(-0.023, -0.002) | 0.024 |  |

| **Supplementary Table S2 Subgroup analysis for the association between SIRI and vitamin K intake.** | | | |
| --- | --- | --- | --- |
| character | β(95% CI) | p | p for interaction |
| Age group | |  | 0.684 |
| [20,45) | 0( 0.000, 0.000) | 0.99 |  |
| [45,60) | 0( 0.000, 0.000) | 0.163 |  |
| [60,) | 0( 0.000, 0.000) | 0.271 |  |
| Gender |  |  | 0.057 |
| Female | 0( 0.000, 0.000) | 0.561 |  |
| Male | 0( 0.000, 0.000) | 0.041 |  |
| Race |  |  | < 0.0001 |
| Non-Hispanic White | 0( 0.000, 0.000) | 0.093 |  |
| Non-Hispanic Black | 0( 0.000, 0.000) | 0.229 |  |
| Mexican American | 0( 0.000, 0.000) | 0.171 |  |
| Other Hispanic | 0( 0.000, 0.000) | 0.084 |  |
| Other Race | 0( 0.000,0.000) | <0.0001 |  |
| BMI group | |  | 0.196 |
| underweight | 0( 0.000, 0.000) | 0.339 |  |
| normal | 0( 0.000, 0.000) | 0.12 |  |
| overweight | 0( 0.000, 0.000) | 0.015 |  |
| obese | 0( 0.000, 0.000) | 0.401 |  |
| Hyperlipidemia | |  | 0.336 |
| yes | 0( 0.000, 0.000) | 0.086 |  |
| no | 0( 0.000, 0.000) | 0.041 |  |
| Diabetes |  |  | 0.761 |
| yes | 0( 0.000, 0.000) | 0.541 |  |
| no | 0( 0.000, 0.000) | 0.042 |  |
| Hypertension | |  | 0.273 |
| yes | 0( 0.000, 0.000) | 0.194 |  |
| no | 0( 0.000, 0.000) | 0.041 |  |

| **Supplementary Table S3 Subgroup analysis for the association between SIIRI and vitamin K intake.** | | | |
| --- | --- | --- | --- |
| character | β(95% CI) | p | p for interaction |
| Age group | |  | 0.979 |
| [20,45) | -0.014(-0.042, 0.013) | 0.294 |  |
| [45,60) | -0.014(-0.034, 0.006) | 0.163 |  |
| [60,) | -0.012(-0.022, -0.002) | 0.021 |  |
| Gender |  |  | 0.085 |
| Female | -0.006(-0.016, 0.003) | 0.194 |  |
| Male | -0.027(-0.051, -0.003) | 0.031 |  |
| Race |  |  | 0.002 |
| Non-Hispanic White | -0.019( -0.035, -0.003) | 0.019 |  |
| Non-Hispanic Black | -0.018( -0.044, 0.009) | 0.192 |  |
| Mexican American | 0.052( -0.025, 0.129) | 0.182 |  |
| Other Hispanic | -0.004( -0.009, 0.001) | 0.103 |  |
| Other Race | -0.072( -0.111, -0.033) | <0.001 |  |
| BMI group | |  | 0.3 |
| underweight | -0.081(-0.178, 0.015) | 0.097 |  |
| normal | -0.009(-0.017, -0.001) | 0.03 |  |
| overweight | -0.041(-0.081, -0.002) | 0.041 |  |
| obese | -0.02(-0.052, 0.012) | 0.221 |  |
| Hyperlipidemia | |  | 0.623 |
| yes | -0.012(-0.024, -0.001) | 0.032 |  |
| no | -0.028(-0.055, 0.000) | 0.048 |  |
| Diabetes |  |  | 0.848 |
| yes | -0.015(-0.054, 0.025) | 0.461 |  |
| no | -0.013(-0.024, -0.002) | 0.019 |  |
| Hypertension | |  | 0.156 |
| yes | -0.032(-0.065, 0.001) | 0.061 |  |
| no | -0.009( -0.018, -0.001) | 0.031 |  |

| **Supplementary Table S4 Subgroup analysis for the association between NLR and vitamin K intake.** | | | |
| --- | --- | --- | --- |
| character | β(95% CI) | p | p for interaction |
| Age group | |  | 0.519 |
| [20,45) | 0( 0.000, 0.000) | 0.812 |  |
| [45,60) | 0( 0.000, 0.000) | 0.091 |  |
| [60,) | 0( 0.000, 0.000) | 0.027 |  |
| Gender |  |  | 0.257 |
| Female | 0( 0.000, 0.000) | 0.447 |  |
| Male | 0( 0.000, 0.000) | 0.018 |  |
| Race |  |  | 0.058 |
| Non-Hispanic White | 0( 0.000, 0.000) | 0.014 |  |
| Non-Hispanic Black | 0( 0.000, 0.000) | 0.746 |  |
| Mexican American | 0( 0.000, 0.000) | 0.382 |  |
| Other Hispanic | 0( 0.000,0.000) | 0.015 |  |
| Other Race | 0( 0.000,0.000) | 0.007 |  |
| BMI group | |  | 0.136 |
| underweight | 0( 0.000, 0.001) | 0.377 |  |
| normal | 0( 0.000, 0.000) | 0.122 |  |
| overweight | 0( 0.000, 0.000) | <0.001 |  |
| obese | 0( 0.000, 0.000) | 0.715 |  |
| Hyperlipidemia | |  | 0.355 |
| yes | 0( 0.000, 0.000) | 0.055 |  |
| no | 0( 0.000, 0.000) | 0.06 |  |
| Diabetes |  |  | 0.511 |
| yes | 0( 0.000, 0.000) | 0.69 |  |
| no | 0( 0.000, 0.000) | 0.016 |  |
| Hypertension | |  | 0.194 |
| yes | 0( 0.000, 0.000) | 0.143 |  |
| no | 0( 0.000, 0.000) | 0.035 |  |

| **Supplementary Table S5 Subgroup analysis for the association between PLR and vitamin K intake.** | | | |
| --- | --- | --- | --- |
| character | β(95% CI) | p | p for interaction |
| Age group | |  | 0.44 |
| [20,45) | -0.001( -0.008, 0.005) | 0.645 |  |
| [45,60) | -0.001( -0.003, 0.000) | 0.143 |  |
| [60,) | -0.003( -0.005, -0.001) | 0.006 |  |
| Gender |  |  | 0.517 |
| Female | -0.002( -0.003, 0.000) | 0.048 |  |
| Male | -0.002( -0.005, 0.000) | 0.096 |  |
| Race |  |  | 0.378 |
| Non-Hispanic White | -0.003( -0.006, -0.001) | 0.015 |  |
| Non-Hispanic Black | 0.004( -0.006, 0.014) | 0.434 |  |
| Mexican American | 0.009( -0.012, 0.029) | 0.399 |  |
| Other Hispanic | -0.001( -0.001,-0.001) | <0.0001 |  |
| Other Race | -0.006( -0.018, 0.006) | 0.295 |  |
| BMI group | |  | 0.418 |
| underweight | 0.008( -0.045, 0.061) | 0.75 |  |
| normal | -0.002( -0.003, 0.000) | 0.051 |  |
| overweight | -0.004( -0.013, 0.005) | 0.393 |  |
| obese | -0.004( -0.008,-0.001) | 0.027 |  |
| Hyperlipidemia | |  | 0.93 |
| yes | -0.002( -0.004, -0.001) | 0.004 |  |
| no | 0( -0.008, 0.009) | 0.966 |  |
| Diabetes |  |  | 0.43 |
| yes | -0.002( -0.008, 0.003) | 0.463 |  |
| no | -0.002( -0.004, 0.000) | 0.017 |  |
| Hypertension | |  | 0.78 |
| yes | -0.002( -0.008, 0.003) | 0.37 |  |
| no | -0.002( -0.003, 0.000) | 0.015 |  |

| **Supplementary Table S6 Subgroup analysis for the association between RAR and vitamin K intake.** | | | |
| --- | --- | --- | --- |
| character | β(95% CI) | p | p for interaction |
| Age group | |  | 0.435 |
| [20,45) | 0( 0.000, 0.000) | 0.777 |  |
| [45,60) | 0( 0.000, 0.000) | 0.143 |  |
| [60,) | 0( 0.000, 0.000) | 0.48 |  |
| Gender |  |  | 0.016 |
| Female | 0( 0.000, 0.000) | 0.057 |  |
| Male | 0( 0.000, 0.000) | 0.5 |  |
| Race |  |  | 0.103 |
| Non-Hispanic White | 0( 0.000, 0.000) | 0.345 |  |
| Non-Hispanic Black | 0( 0.000, 0.000) | 0.157 |  |
| Mexican American | 0( 0.000, 0.000) | 0.397 |  |
| Other Hispanic | 0( 0.000, 0.000) | <0.0001 |  |
| Other Race | 0( 0.000, 0.000) | 0.23 |  |
| BMI group | |  | 0.352 |
| underweight | 0( 0.000, 0.001) | 0.057 |  |
| normal | 0( 0.000, 0.000) | 0.296 |  |
| overweight | 0( 0.000, 0.000) | 0.719 |  |
| obese | 0( 0.000, 0.000) | 0.233 |  |
| Hyperlipidemia | |  | 0.405 |
| yes | 0( 0.000, 0.000) | 0.116 |  |
| no | 0( 0.000, 0.000) | 0.677 |  |
| Diabetes |  |  | 0.202 |
| yes | 0( 0.000, 0.000) | 0.192 |  |
| no | 0( 0.000, 0.000) | 0.334 |  |
| Hypertension | |  | 0.213 |
| yes | 0( 0.000, 0.000) | 0.167 |  |
| no | 0( 0.000, 0.000) | 0.389 |  |

| **Supplementary Table S7 Subgroup analysis for the association between ferritin and vitamin K intake.** | | | |
| --- | --- | --- | --- |
| character | β(95% CI) | p | p for interaction |
| Age group | |  | 0.256 |
| [20,45) | -0.002( -0.013, 0.009) | 0.73 |  |
| [45,60) | 0.023( -0.022, 0.067) | 0.312 |  |
| [60,) | -0.011( -0.021, -0.002) | 0.022 |  |
| Gender |  |  | 0.259 |
| Female | -0.004( -0.011, 0.003) | 0.284 |  |
| Male | -0.005( -0.045, 0.036) | 0.81 |  |
| Race |  |  | 0.77 |
| Non-Hispanic White | -0.001( -0.012, 0.009) | 0.792 |  |
| Non-Hispanic Black | 0.014( -0.025, 0.052) | 0.476 |  |
| Mexican American | -0.008( -0.067, 0.052) | 0.79 |  |
| Other Hispanic | -0.01( -0.046, 0.027) | 0.592 |  |
| Other Race | -0.016( -0.072, 0.040) | 0.576 |  |
| BMI group | |  | 0.607 |
| underweight | 0.002( -0.060, 0.064) | 0.938 |  |
| normal | 0.004( -0.012, 0.020) | 0.618 |  |
| overweight | -0.013( -0.046, 0.019) | 0.416 |  |
| obese | 0.004( -0.012, 0.020) | 0.615 |  |
| Hyperlipidemia | |  | 0.063 |
| yes | -0.005( -0.014, 0.003) | 0.227 |  |
| no | 0.023( -0.002, 0.048) | 0.074 |  |
| Diabetes |  |  | 0.01 |
| yes | -0.008( -0.020, 0.004) | 0.199 |  |
| no | 0.009( -0.005, 0.022) | 0.197 |  |
| Hypertension | |  | 0.016 |
| yes | -0.009( -0.022, 0.004) | 0.172 |  |
| no | 0.012( -0.003, 0.026) | 0.119 |  |

| **Supplementary Table S8 Subgroup analysis for the association between hs-CRP and vitamin K intake.** | | | |
| --- | --- | --- | --- |
| character | β(95% CI) | p | p for interaction |
| Age group | |  | 0.928 |
| [20,45) | 0( 0.000, 0.001) | 0.697 |  |
| [45,60) | -0.001(-0.003, 0.001) | 0.413 |  |
| [60,) | 0( 0.000, 0.001) | 0.611 |  |
| Gender |  |  | 0.075 |
| Female | 0(-0.001, 0.000) | 0.914 |  |
| Male | 0(-0.001, 0.001) | 0.48 |  |
| Race |  |  | 0.162 |
| Non-Hispanic White | 0( 0.000, 0.001) | 0.525 |  |
| Non-Hispanic Black | -0.002(-0.004,-0.001) | 0.008 |  |
| Mexican American | 0.001(-0.002, 0.003) | 0.577 |  |
| Other Hispanic | 0(-0.002, 0.002) | 0.955 |  |
| Other Race | 0(-0.001, 0.001) | 0.58 |  |
| BMI group | |  | 0.212 |
| underweight | -0.009( -0.029, 0.011) | 0.26 |  |
| normal | 0( 0.000, 0.001) | 0.345 |  |
| overweight | 0(-0.001, 0.002) | 0.633 |  |
| obese | 0(-0.001, 0.000) | 0.397 |  |
| Hyperlipidemia | |  | < 0.0001 |
| yes | 0( 0.000, 0.001) | 0.377 |  |
| no | -0.001(-0.002, 0.000) | 0.041 |  |
| Diabetes |  |  | 0.237 |
| yes | 0(-0.002, 0.001) | 0.488 |  |
| no | 0( 0.000, 0.001) | 0.813 |  |
| Hypertension | |  | 0.984 |
| yes | 0(-0.001, 0.001) | 0.954 |  |
| no | 0(-0.001, 0.001) | 0.982 |  |

| **Supplementary Table S9 Subgroup analysis for the association between WBC and vitamin K intake.** | | | |
| --- | --- | --- | --- |
| character | β(95% CI) | p | p for interaction |
| Age group | |  | 0.923 |
| [20,45) | 0(-0.001, 0.000) | 0.158 |  |
| [45,60) | 0( 0.000, 0.000) | 0.332 |  |
| [60,) | 0( 0.000, 0.000) | 0.205 |  |
| Gender |  |  | 0.029 |
| Female | 0( 0.000, 0.000) | 0.568 |  |
| Male | 0( 0.000, 0.000) | 0.016 |  |
| Race |  |  | 0.118 |
| Non-Hispanic White | 0( 0.000, 0.000) | 0.038 |  |
| Non-Hispanic Black | -0.001(-0.001, 0.000) | 0.065 |  |
| Mexican American | 0( 0.000, 0.001) | 0.47 |  |
| Other Hispanic | 0( 0.000, 0.000) | 0.9 |  |
| Other Race | 0(-0.001, 0.000) | 0.061 |  |
| BMI group | |  | 0.193 |
| underweight | 0(-0.001, 0.001) | 0.809 |  |
| normal | 0( 0.000, 0.000) | 0.177 |  |
| overweight | 0(-0.001, 0.000) | 0.039 |  |
| obese | 0( 0.000, 0.000) | 0.501 |  |
| Hyperlipidemia | |  | 0.448 |
| yes | 0( 0.000, 0.000) | 0.101 |  |
| no | 0( 0.000, 0.000) | 0.45 |  |
| Diabetes |  |  | 0.462 |
| yes | 0( 0.000, 0.000) | 0.892 |  |
| no | 0( 0.000, 0.000) | 0.101 |  |
| Hypertension | |  | 0.239 |
| yes | 0(-0.001, 0.000) | 0.116 |  |
| no | 0( 0.000, 0.000) | 0.261 |  |

| **Supplementary Table S10 Subgroup analysis for the association between Neut and vitamin K intake.** | | | |
| --- | --- | --- | --- |
| character | β(95% CI) | p | p for interaction |
| Age group | |  | 0.891 |
| [20,45) | 0( 0.000, 0.000) | 0.346 |  |
| [45,60) | 0( 0.000, 0.000) | 0.233 |  |
| [60,) | 0( 0.000, 0.000) | 0.286 |  |
| Gender |  |  | 0.026 |
| Female | 0( 0.000, 0.000) | 0.719 |  |
| Male | 0( 0.000, 0.000) | 0.019 |  |
| Race |  |  | 0.079 |
| Non-Hispanic White | 0( 0.000, 0.000) | 0.037 |  |
| Non-Hispanic Black | 0( 0.000, 0.000) | 0.132 |  |
| Mexican American | 0( 0.000, 0.001) | 0.458 |  |
| Other Hispanic | 0( 0.000, 0.000) | 0.505 |  |
| Other Race | 0(-0.001, 0.000) | 0.012 |  |
| BMI group | |  | 0.209 |
| underweight | 0(-0.001, 0.001) | 0.729 |  |
| normal | 0( 0.000, 0.000) | 0.102 |  |
| overweight | 0(-0.001, 0.000) | 0.024 |  |
| obese | 0( 0.000, 0.000) | 0.914 |  |
| Hyperlipidemia | |  | 0.474 |
| yes | 0( 0.000, 0.000) | 0.076 |  |
| no | 0( 0.000, 0.000) | 0.354 |  |
| Diabetes |  |  | 0.829 |
| yes | 0( 0.000, 0.000) | 0.868 |  |
| no | 0( 0.000, 0.000) | 0.068 |  |
| Hypertension | |  | 0.218 |
| yes | 0( 0.000, 0.000) | 0.163 |  |
| no | 0( 0.000, 0.000) | 0.167 |  |

| **Supplementary Table S11 Subgroup analysis for the association between Lym and vitamin K intake.** | | | |
| --- | --- | --- | --- |
| character | β(95% CI) | p | p for interaction |
| Age group | |  | 0.664 |
| [20,45) | 0( 0.000, 0.000) | 0.203 |  |
| [45,60) | 0( 0.000, 0.000) | 0.737 |  |
| [60,) | 0( 0.000, 0.000) | 0.273 |  |
| Gender |  |  | 0.304 |
| Female | 0( 0.000, 0.000) | 0.628 |  |
| Male | 0( 0.000, 0.000) | 0.098 |  |
| Race |  |  | 0.236 |
| Non-Hispanic White | 0( 0.000, 0.000) | 0.16 |  |
| Non-Hispanic Black | 0(-0.001, 0.000) | 0.155 |  |
| Mexican American | 0( 0.000, 0.000) | 0.938 |  |
| Other Hispanic | 0( 0.000, 0.000) | <0.0001 |  |
| Other Race | 0( 0.000, 0.000) | 0.212 |  |
| BMI group | |  | 0.246 |
| underweight | 0(-0.001, 0.000) | 0.032 |  |
| normal | 0( 0.000, 0.000) | 0.6 |  |
| overweight | 0( 0.000, 0.000) | 0.59 |  |
| obese | 0( 0.000, 0.000) | 0.342 |  |
| Hyperlipidemia | |  | 0.602 |
| yes | 0( 0.000, 0.000) | 0.308 |  |
| no | 0( 0.000, 0.000) | 0.935 |  |
| Diabetes |  |  | 0.183 |
| yes | 0( 0.000, 0.000) | 0.92 |  |
| no | 0( 0.000, 0.000) | 0.316 |  |
| Hypertension | |  | 0.443 |
| yes | 0( 0.000, 0.000) | 0.157 |  |
| no | 0( 0.000, 0.000) | 0.945 |  |

| **Supplementary Table S12 Subgroup analysis for the association between MO and vitamin K intake.** | | | |
| --- | --- | --- | --- |
| character | β(95% CI) | p | p for interaction |
| Age group | |  | 0.58 |
| [20,45) | 0( 0.000, 0.000) | 0.438 |  |
| [45,60) | 0( 0.000, 0.000) | 0.218 |  |
| [60,) | 0( 0.000, 0.000) | 0.716 |  |
| Gender |  |  | 0.097 |
| Female | 0( 0.000, 0.000) | 0.851 |  |
| Male | 0( 0.000, 0.000) | 0.122 |  |
| Race |  |  | 0.001 |
| Non-Hispanic White | 0( 0.000, 0.000) | 0.65 |  |
| Non-Hispanic Black | 0( 0.000, 0.000) | 0.524 |  |
| Mexican American | 0( 0.000, 0.000) | 0.38 |  |
| Other Hispanic | 0( 0.000, 0.000) | 0.086 |  |
| Other Race | 0( 0.000, 0.000) | <0.001 |  |
| BMI group | |  | 0.423 |
| underweight | 0( 0.000, 0.000) | 0.204 |  |
| normal | 0( 0.000, 0.000) | 0.641 |  |
| overweight | 0( 0.000, 0.000) | 0.777 |  |
| obese | 0( 0.000, 0.000) | 0.239 |  |
| Hyperlipidemia | |  | 0.821 |
| yes | 0( 0.000, 0.000) | 0.382 |  |
| no | 0( 0.000, 0.000) | 0.205 |  |
| Diabetes |  |  | 0.184 |
| yes | 0( 0.000, 0.000) | 0.825 |  |
| no | 0( 0.000, 0.000) | 0.299 |  |
| Hypertension | |  | 0.964 |
| yes | 0( 0.000, 0.000) | 0.771 |  |
| no | 0( 0.000, 0.000) | 0.117 |  |

| **Supplementary Table S13 Subgroup analysis for the association between EOS and vitamin K intake.** | | | |
| --- | --- | --- | --- |
| character | β(95% CI) | p | p for interaction |
| Age group | |  | 0.366 |
| [20,45) | 0( 0.000, 0.000) | 0.025 |  |
| [45,60) | 0( 0.000, 0.000) | 0.175 |  |
| [60,) | 0( 0.000, 0.000) | 0.066 |  |
| Gender |  |  | 0.158 |
| Female | 0( 0.000, 0.000) | 0.082 |  |
| Male | 0( 0.000, 0.000) | 0.029 |  |
| Race |  |  | 0.055 |
| Non-Hispanic White | 0( 0.000, 0.000) | 0.015 |  |
| Non-Hispanic Black | 0( 0.000, 0.000) | 0.416 |  |
| Mexican American | 0( 0.000, 0.000) | 0.21 |  |
| Other Hispanic | 0( 0.000, 0.000) | 0.153 |  |
| Other Race | 0( 0.000, 0.000) | 0.006 |  |
| BMI group | |  | 0.037 |
| underweight | 0( 0.000, 0.000) | 0.512 |  |
| normal | 0( 0.000, 0.000) | 0.02 |  |
| overweight | 0( 0.000, 0.000) | 0.002 |  |
| obese | 0( 0.000, 0.000) | 0.086 |  |
| Hyperlipidemia | |  | 0.268 |
| yes | 0( 0.000, 0.000) | 0.01 |  |
| no | 0( 0.000, 0.000) | 0.946 |  |
| Diabetes |  |  | 0.394 |
| yes | 0( 0.000, 0.000) | 0.114 |  |
| no | 0( 0.000, 0.000) | 0.018 |  |
| Hypertension | |  | 0.063 |
| yes | 0( 0.000, 0.000) | 0.005 |  |
| no | 0( 0.000, 0.000) | 0.017 |  |

| **Supplementary Table S14 Subgroup analysis for the association between BAS and vitamin K intake.** | | | |
| --- | --- | --- | --- |
| character | β(95% CI) | p | p for interaction |
| Age group | |  | 0.451 |
| [20,45) | 0( 0.000, 0.000) | 0.505 |  |
| [45,60) | 0( 0.000, 0.000) | 0.168 |  |
| [60,) | 0( 0.000, 0.000) | 0.776 |  |
| Gender |  |  | 0.054 |
| Female | 0( 0.000, 0.000) | 0.44 |  |
| Male | 0( 0.000, 0.000) | 0.044 |  |
| Race |  |  | 0.544 |
| Non-Hispanic White | 0( 0.000, 0.000) | 0.843 |  |
| Non-Hispanic Black | 0( 0.000,0.000) | 0.557 |  |
| Mexican American | 0( 0.000, 0.000) | 0.233 |  |
| Other Hispanic | 0( 0.000, 0.000) | 0.006 |  |
| Other Race | 0( 0.000,0.000) | 0.629 |  |
| BMI group | |  | 0.048 |
| underweight | 0( 0.000, 0.000) | 0.048 |  |
| normal | 0( 0.000,0.000) | 0.644 |  |
| overweight | 0( 0.000, 0.000) | 0.036 |  |
| obese | 0( 0.000, 0.000) | 0.672 |  |
| Hyperlipidemia | |  | 0.172 |
| yes | 0( 0.000, 0.000) | 0.602 |  |
| no | 0( 0.000, 0.000) | 0.32 |  |
| Diabetes |  |  | 0.005 |
| yes | 0( 0.000, 0.000) | 0.003 |  |
| no | 0( 0.000, 0.000) | 0.466 |  |
| Hypertension | |  | 0.722 |
| yes | 0( 0.000, 0.000) | 0.892 |  |
| no | 0( 0.000, 0.000) | 0.861 |  |

Abbreviations: SII, systemic immune-inflammation index; SIRI, systemic inflammation response index; SIIRI, systemic immune-inflammation response index; NLR, neutrophil-to-lymphocyte ratio; PLR, platelet-to-lymphocyte ratio; RAR, red blood cell distribution width-to-albumin ratio; hs-CRP, high-sensitivity C- reactive protein; WBC, white blood cell; Neut, neutrophil; Lym, lymphocyte; MO, monocyte; EOS, eosinophil; BAS, basophil.
