# Supplementary material for: Associations between vitamin K and systemic immune and inflammation biomarkers: a population-based study from the NHANES (2007–2020)
Source: Front Nutr. 2025 Jul 11;12:1625209. doi: 10.3389/fnut.2025.1625209 (PMC12289624; doi:10.3389/fnut.2025.1625209)
Supplement: Supplementary file 4 [file Image_1.pdf]

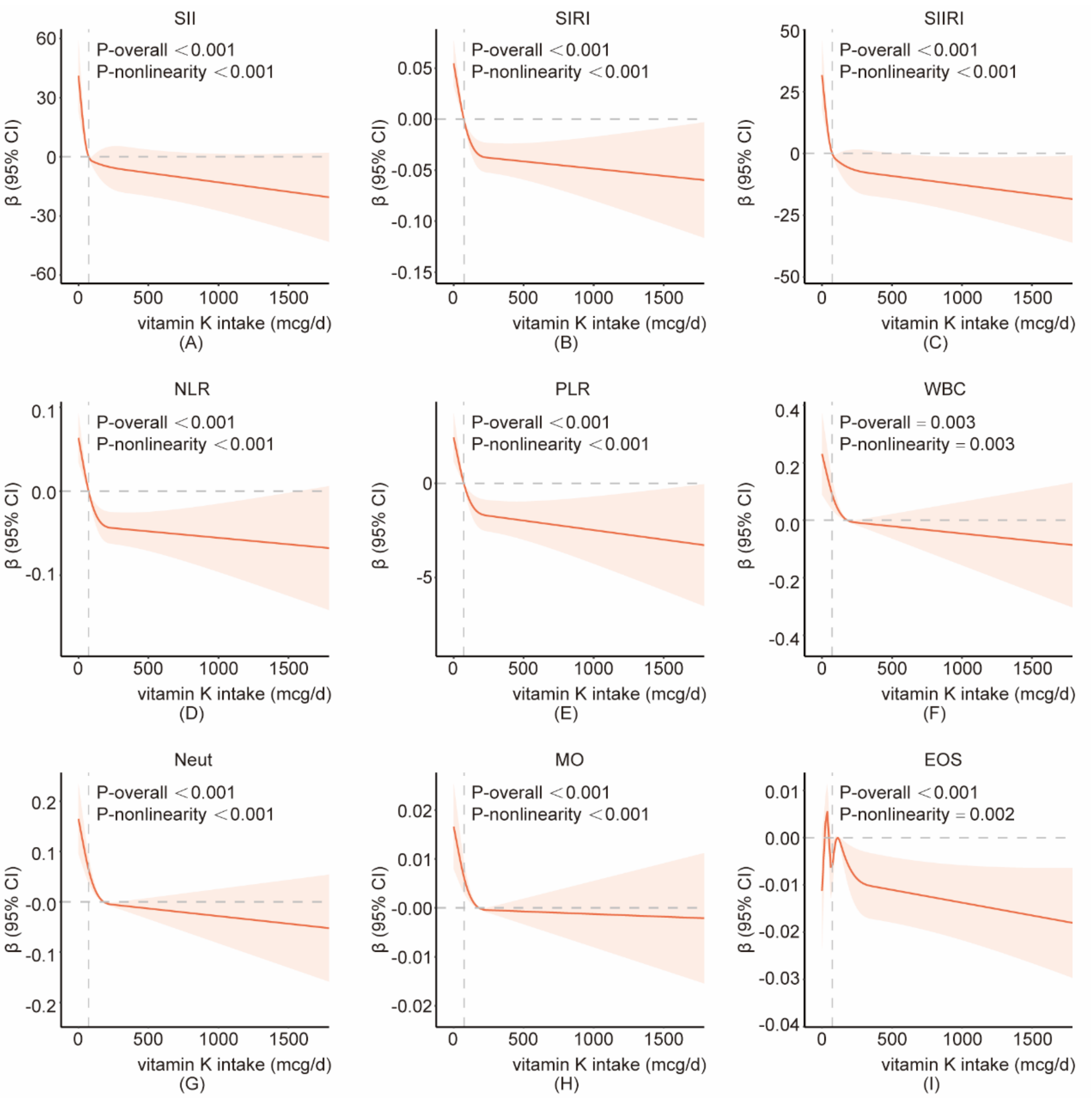

Supplementary Figure S1 The restricted cubic spline between vitamin K and SII(A), SIRI(B), SIIRI(C), NLR(D), PLR(E), WBC(F), Neut(G), MO(H), EOS(I). Adjusted for age, gender, race, family poverty income ratio, educational level, BMI, smoking status, alcohol consumption, vigorous recreational activity, dietary inflammatory index, hyperlipidemia, hypertension, and diabetes. The red curve represents the fitted RCS curve. The light red shaded area indicates the 95% confidence interval for the β estimates. The vitamin K intake corresponding to the vertical dotted line is 72 mcg/d, and this value represents the median.

Abbreviations: SII, systemic immune-inflammation index; SIRI, systemic inflammation response index; SIIRI, systemic immune-inflammation response index; NLR, neutrophil-to-lymphocyte ratio; PLR, platelet-to-lymphocyte ratio; WBC, white blood cell; Neut, neutrophil; Lym, lymphocyte; MO, monocyte; EOS, eosinophil
